# Zn and Ni complexes of pyridine-2,6-di­carboxyl­ates: crystal field stabilization matters!

**DOI:** 10.1107/S2056989019007461

**Published:** 2019-05-31

**Authors:** Marius Kremer, Ulli Englert

**Affiliations:** a Institut für Anorganische Chemie, RWTH Aachen University, Landoltweg 1, 52074 Aachen, Germany

**Keywords:** crystal structure, crystal field stabilization, pyridine di­carb­oxy­lic acids, polydentate ligands, coordination polymers, Ni^II^ complexes, Zn^II^ complexes

## Abstract

Six reaction products of Zn^II^ and Ni^II^ with pyridine-2,6-di­carb­oxy­lic acid (H_2_Lig^1^), 4-chloro­pyridine-2,6-di­carb­oxy­lic acid (H_2_Lig^2^) and 4-hy­droxy­pyridine-2,6-di­carb­oxy­lic acid (H_2_Lig^3^) are used to pinpoint the structural consequences of crystal field stabilization by an incomplete *d* shell. The pseudo-octa­hedral Zn^II^ coordination sphere in bis­(6-carb­oxy­picolinato)zinc(II) trihydrate, [Zn(C_7_H_4_NO_4_)_2_]·3H_2_O or [Zn(HLig^1^)_2_]·3H_2_O, (**1**), is significantly less regular than that about Ni^II^ in the isostructural compound bis­(6-carb­oxy­picolinato)nickel(II) trihydrate, [Ni(C_7_H_4_NO_4_)_2_]·3H_2_O or [Ni(HLig^1^)_2_]·3H_2_O, (**2**). The Zn^II^ complexes poly[(4-chloro­pyridine-2,6-di­carboxyl­ato)zinc(II)], [Zn(C_7_H_2_ClNO_4_)]_*n*_ or [Zn(Lig^2^)]_*n*_, (**3**), and poly[[(4-hy­droxy­pyridine-2,6-di­carboxyl­ato)zinc(II)] monohydrate], {[Zn(C_7_H_3_NO_5_)]·H_2_O}_*n*_ or {[Zn(Lig^3^)]·H_2_O}_*n*_, (**4**), represent two-dimensional coordination polymers with chelating and bridging pyridine-2,6-di­carboxyl­ate ligands in which the coordination polyhedra about the central cations cannot be associated with any regular shape; their coordination environments range between trigonal–bipyramidal and square-pyramidal geometries. In contrast, the corresponding adducts of the diprotonated ligands to Ni^II^, namely tri­aqua­(4-chloro­pyridine-2,6-di­carboxyl­ato)nickel(II), [Ni(C_7_H_2_ClNO_4_)(H_2_O)_3_] or [NiLig^2^(OH_2_)_3_)], (**5**), and tri­aqua­(4-hy­droxy­pyridine-2,6-di­carboxyl­ato)nickel(II) 1.7-hydrate, [Ni(C_7_H_3_NO_5_)(H_2_O)_3_]·1.7H_2_O or [NiLig^3^(OH_2_)_3_)]·1.7H_2_O, (**6**), feature rather regular octa­hedral coordination spheres about the transition-metal cations, thus precluding the formation of analogous extended structures.

## Abstract 2

Different electron configurations for Zn^II^ (*d* ^10^) and Ni^II^ (*d* ^8^) lead to less regular coordination environments for the former: Zn^II^ adopts strongly distorted octa­hedral or even fivefold coordination spheres whereas Ni^II^ favours rather regular octa­hedra. As a consequence, only the more flexible zinc cations may act as nodes in two-dimensional extended structures.

## Chemical context   

**Figure 1:**
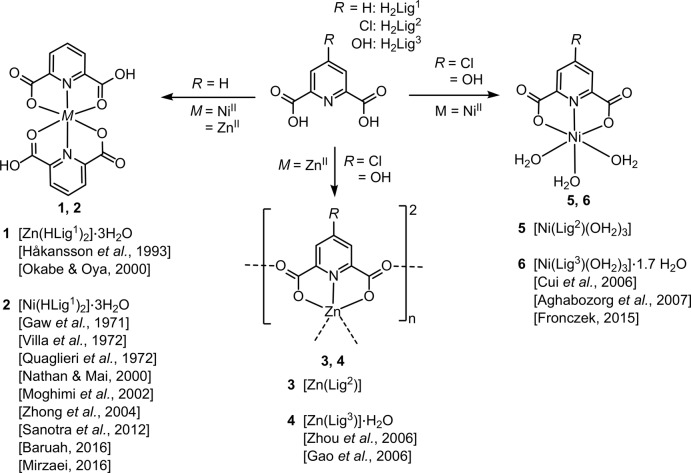
Compilation of the structural characterizations performed in the context of this work and of previous literature. References: **1**: Håkansson *et al.* (1993 ▸); Okabe & Oya (2000 ▸); **2**: Gaw *et al.* (1971 ▸); Villa *et al.* (1972 ▸); Quaglieri *et al.* (1972 ▸); Nathan & Mai (2000 ▸); Moghimi *et al.* (2002 ▸); Zhong *et al.* (2004 ▸); Sanotra *et al.* (2012 ▸); Baruah (2016 ▸); Mirzaei (2016 ▸); **4**: Zhou *et al.* (2006 ▸); Gao *et al.* (2006 ▸); **6**: Cui *et al.* (2006 ▸); Aghabozorg *et al.* (2007 ▸); Fronczek (2015 ▸).

Pyridine-2,6-di­carb­oxy­lic acid (H_2_Lig^1^, Fig. 1 ▸) represents a popular building block in coordination chemistry: the Cambridge Structural Database (CSD; Groom *et al.*, 2016 ▸) comprises 1404 structurally characterized metal complexes of this ligand. Its 4-chloro (H_2_Lig^2^) and 4-hy­droxy (H_2_Lig^3^) derivatives have been employed less frequently, with only 10 and 136 entries, respectively, in the CSD. We have investigated these three pyridine-2,6-di­carb­oxy­lic acids, Lig^1^–Lig^3^, in a comprehensive study of their complexes with Ni^II^ and Zn^II^. We focus on these cations for the following reasons: (*a*) According to the widely used compilation of Shannon (1976 ▸), Ni^II^ and Zn^II^ adopt comparable ionic radii of 0.69 and 0.74 Å, respectively, in their six-coordinated complexes. Alternative divalent cations might be Mn^II^ and Cu^II^; the former is associated with a significantly larger ionic radius, the latter is notoriously Jahn–Teller distorted. (*b*) For Ni^II^ and Zn^II^, undistorted octa­hedral complexes can, in principle, be expected. Crystal field stabilization energy for Ni^II^ results in a clear preference for regular coordination, with the fully occupied *t*_2*g*_ orbitals directed in-between and the only half-occupied *e_g_* orbitals towards the octa­hedrally disposed ligands. No such electronic effects are expected for the *d* ^10^-configured Zn^II^ ion: in this case, a regular coordination is neither preferred nor excluded. We use our structural results on the Ni^II^ and Zn^II^ derivatives compiled in Fig. 1 ▸ to pinpoint the different coordination behaviour of these divalent cations; Fig. 1 ▸ also reports previous results by other authors that have been obtained for the same compounds and, to the best of our knowledge, have never been put into a common context.
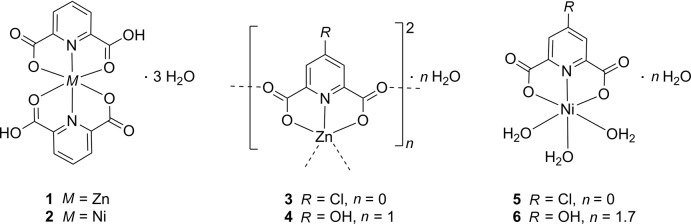


## Structural comparison   

**Mononuclear bis­(6-carb­oxy­picolinato) complexes**

**Figure 2:**
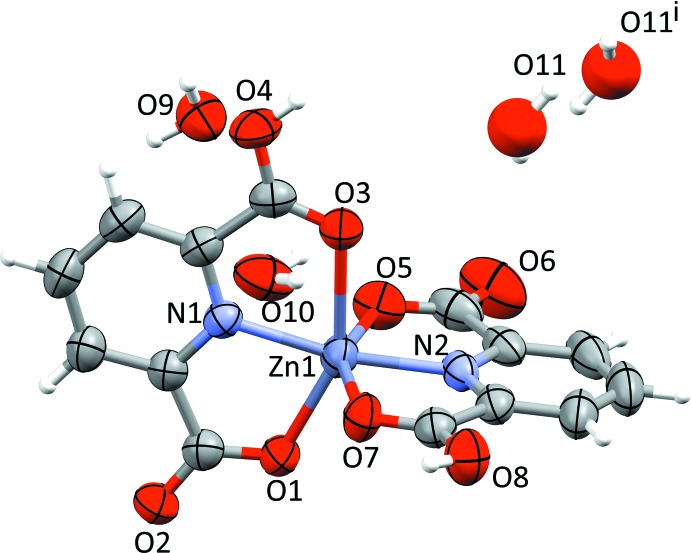
Displacement ellipsoid plot (Macrae *et al.*, 2006 ▸) of the asymmetric unit of **1**. Sites of minor occupancy for O11 have been omitted. Displacement ellipsoids are drawn at the 70% probability level and H atoms are shown as spheres of arbitrary radii.

We start our comparison between Ni^II^ and Zn^II^ coordination with their mononuclear complexes with two equivalents of monodeprotonated Lig^1^. The resulting products **1** and **2** have previously been structurally characterized and are isostructural. Their asymmetric unit contains a complex mol­ecule and three mol­ecules of water; one of the latter is disordered over three neighbouring and mutually exclusive positions. The previous studies of **1** (Håkansson *et al.*, 1993 ▸; Okabe & Oya, 2000 ▸) agree with our structural model as far as the bis­(Hlig^1^) complex is concerned, but the three water mol­ecules were treated as ordered; both studies find an equivalent displacement parameter of 0.28 Å^2^ for one of the water sites, clearly excessive when compared to all other displacement parameters in the structure. A displacement ellipsoid plot of the [Zn(HLig^1^)_2_] complex is shown in Fig. 2 ▸.

The three ligand functionalities differ significantly in their bond lengths to the six-coordinated metal cation (Table 1 ▸): the shortest bonds are subtended by the pyridine N atoms, followed by the distances between Zn^II^ and an oxygen atom of the deprotonated carboxyl­ato groups. The O atoms of the carb­oxy­lic acid moieties represent the most distant coordination partners. Our assignment of negatively charged carboxyl­ato and neutral carb­oxy­lic acid moieties matches the assignment of local electron-density maxima close to the latter; the positional parameters for the thus located H atoms could be freely refined. Each of these hy­droxy H atoms is engaged in a short hydrogen bond to one of the well-ordered water mol­ecules. Our structure model for compound **2** is very similar to that for the isostructural **1**; distances and angles are compiled in Table 2 ▸. Those references to previous reports of the crystal structure of **2** that agree with our inter­pretation are compiled in Fig. 1 ▸. We here also mention two dissenting opinions: Wang *et al.* (2004 ▸) indexed their diffraction patterns with the same unit cell as we used but inter­preted the electron density as [Ni(Lig^1^)_2_]·2H_3_O·2H_2_O, *i.e*. as the bis­(oxonium) salt of a dianionic nickelate. We doubt this protonation pattern, not only because of the alleged presence of strongly acidic oxonium ions next to carboxyl­ate but also because this alternative structure model comes with short inter-oxygen contacts of *ca* 2.5 Å without any proton in between. A rather recent compilation of related structures (Mirzaei *et al.*, 2014 ▸) refers to **2** as [Ni(HLig^1^)_2_]·H_3_O·2H_2_O, without further explanation concerning the unbalanced charge; the reported unit cell corresponds to that found by us and all consenting authors in Fig. 1 ▸.

After discussing the individual bis-ligand complexes **1** and **2**, we come back to the principal aim of our comparison: despite the strict isotypism between these structures, which even extends to the disorder in the co-crystallized water mol­ecules, the coordination spheres about Zn^II^ in **1** and Ni^II^ in **2** differ significantly. The numerical values of bond lengths and angles compiled in Tables 1 ▸ and 2 ▸ reflect a more regular coordination polyhedron for the crystal-field-stabilized nickel ion. According to classical crystal field theory, the pseudo-octa­hedrally arranged coordinating N and O atoms avoid the electron density associated with the fully occupied *t*_2*g*_ orbitals in the nickel cation with electron configuration *d* ^8^. No such effect is observed for the significantly more distorted coordination about the *d* ^10^-configured Zn^II^.

**Figure 3:**
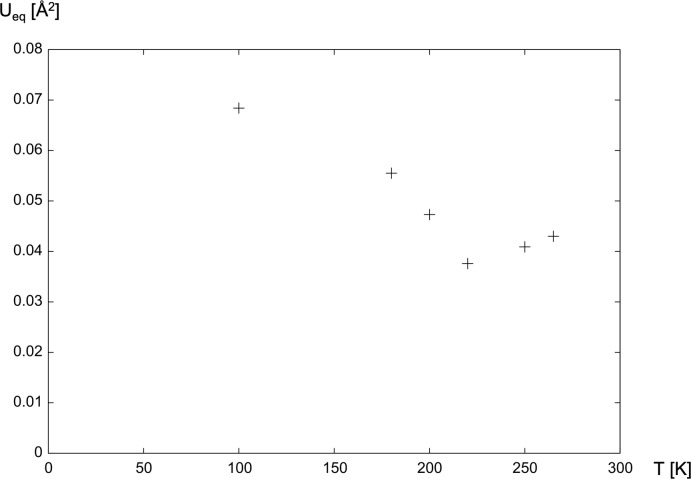
Average *U*_eq_ values for the atoms in the [Zn(HLig^1^)_2_] complex mol­ecule as a function of temperature; *U*_eq_ values for the O atoms in the co-crystallized water mol­ecules were not taken into account.

We finish the discussion of **1** and **2** by explaining our data-collection temperatures: Upon cooling to low temperature, complexes **1** and **2** undergo a reversible phase transition to a larger unit cell. Despite several attempts at different temperatures and cooling rates, we have not been able to completely index the low-temperature diffraction pattern, neither assuming single crystals nor twins. The most promising indexing attempt suggested a non-centrosymmetric body-centered unit cell with four independent complex mol­ecules in the asymmetric unit. Such a low-temperature phase cannot be traced back to a single *t* or *k* type phase transition; rather, it requires a combination of both (Müller, 2013 ▸). In view of the incompletely indexed diffraction pattern, the observed twinning and the large asymmetric unit after the phase transition, we have not been able to deduce a fully satisfactory structural model for the low-temperature phase. In order to establish the transition temperature, we have collected intensity data for **1** as a function of temperature. The temperature dependence of the average *U*_eq_ values for the atoms in the complex mol­ecule is depicted in Fig. 3 ▸.

Based on this relationship and on the fact that it could be satisfactorily indexed, we decided to use the intensity data set collected at 220 K for the structure refinement of **1**. Only data collected at room temperature, at 250 K and a tentative data set at 100 K were available for **2**; our structure refinement is based on the 250 K data.

**Extended coordination networks of 4-substituted di­carboxyl­ato pyridine ligands with Zn^II^**

**Figure 4:**
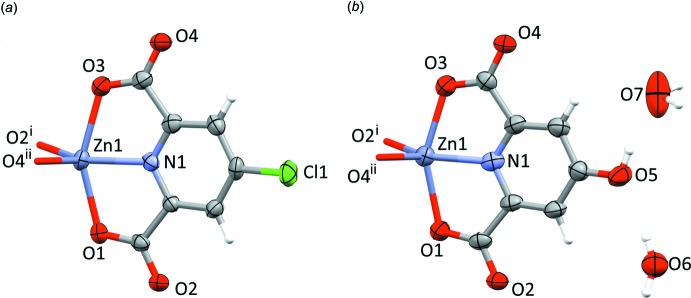
Displacement ellipsoid plots (90% probability, (Macrae *et al.*, 2006 ▸)) of the extended asymmetric unit for (*a*) **3** and (*b*) **4**; H atoms are shown as spheres of arbitrary radii. Symmetry codes: (i) *y*, 2 − *x*, 1 − *z*; (ii) *y*, 1 − *x*, 1 − *z*.

The reaction products of ZnCl_2_ with H_2_Lig^2^ and H_2_Lig^3^ in aqueous solution are isostructural and represent two-dimensional extended structures extending parallel to (001). The asymmetric unit of **3** contains a single formula unit of Zn(Lig^2^) and is depicted in Fig. 4 ▸ *a*; for easier comparison, an analogous representation for the closely related compound **4** is shown in Fig. 4 ▸ *b*.

**Figure 5:**
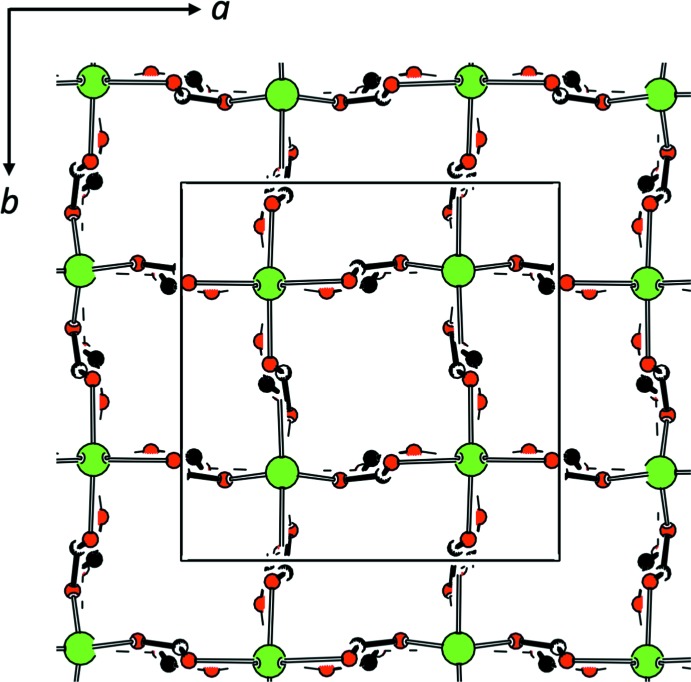
Projection of the unit cell of **3** (Spek, 2009 ▸); only atoms contributing to the extended connectivity in the (001) plane have been included.

One might intuitively associate the coordination about the Zn^II^ cation with a trigonal bipyramid, with O1 and O3 as the axial substituents, but the angle O2^i^—Zn1—N1 [symmetry code: (i) *y*, 2 − *x*, 1 − *z*] also amounts to a relatively large value of 139.20 (10)° (Table 3 ▸). A qu­anti­tative analysis (Holmes, 1984 ▸) places the five-coordination about Zn^II^ almost half-way (48.6%) along a Berry pseudo-rotation coordinate from trigonal–bipyramidal (idealized point group *D*_3*h*_) to square-pyramidal (idealized point group *C*_4*v*_). The alternative *τ* descriptor for fivefold coordination (Addison *et al.*, 1984 ▸) adopts values of 0.20 for **3** and 0.27 for **4** and thus suggests describing the coordination polyhedra about the divalent cations as distorted square-pyramidal (*τ* = 0 for ideal square-pyramidal coordination). The Zn(Lig^2^) units arrange about the 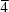 axes in the achiral, non-centrosymmetric space group *P* 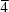
2_1_ *c*. Fig. 5 ▸ shows a projection of the unit cell in which only those atoms contributing to the extended connectivity of a {4,4} net have been included. The shortest secondary inter­action in **3** is a halogen contact, with Cl1⋯O1(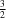 − *y*, 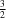 − *x*, 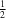 + *z*) = 3.036 (2) Å.

Complex **4** crystallizes in the same space group as **3**, with comparable lattice parameters and similar Zn^II^ coordination (Fig. 4 ▸ *b*, Table 4 ▸). In addition to a Zn(Lig^3^) moiety, its asymmetric unit contains two water mol­ecules on twofold rotation axes; the compound therefore is a monohydrate. The co-crystallized water mol­ecules occupy the twofold axes associated with Wyckoff positions 4*c* and 4*d*. These water mol­ecules subtend short hydrogen bonds with the hy­droxy group of Lig^3^.

**Mononuclear complexes of 4-substituted di­carboxyl­ato pyridine ligands with Ni^II^**

**Figure 6:**
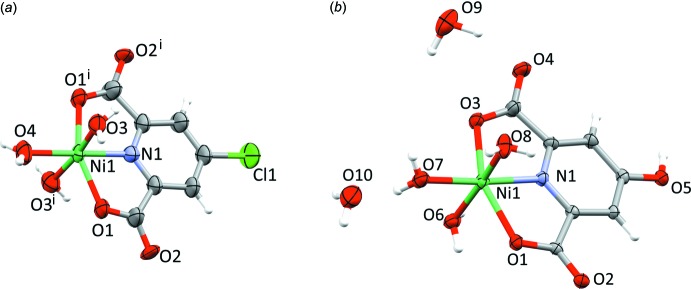
Displacement ellipsoid plots (70% probability, Macrae *et al.*, 2006 ▸) of the asymmetric unit for (*a*) **5** and (*b*) **6**; H atoms are shown as spheres of arbitrary radii. Symmetry code: (i) 1 − *x*, 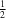 − *y*, *z*.

In contrast to the low-symmetry five-coordinated moieties Zn(Lig) (Lig = Lig^2^, Lig^3^) which act as building blocks for the extended structures of **3** and **4**, coordination of the same ligands to Ni^II^ results in the mononuclear pseudo­octa­hedral complexes **5** and **6**. Complex **5** crystallizes in the tetra­gonal space group *I*4_1_ *a*, with the complex mol­ecule located on a twofold rotation axis. With the exception of the intra-ligand angle O1—Ni1—O1^i^ [symmetry code: (i) −*x* + 1, −*y* + 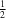, *z*], the coordination sphere about the transition-metal cation corresponds to a rather regular octa­hedron (Fig. 6 ▸ *a*, Table 5 ▸).

Complex **6** is a hydrate; its water content is explained in more detail in the *Refinement* section. The complex mol­ecule [NiLig^2^(OH_2_)_3_)] (Fig. 6 ▸ *b*, Table 6 ▸) adopts a very similar geometry to the Cl-substituted compound **5**. The analogous mononuclear derivative [NiLig^1^(OH_2_)_3_)] has been structurally characterized by Li & Du (2015 ▸).

**Inter­molecular inter­actions**

**Figure 7:**
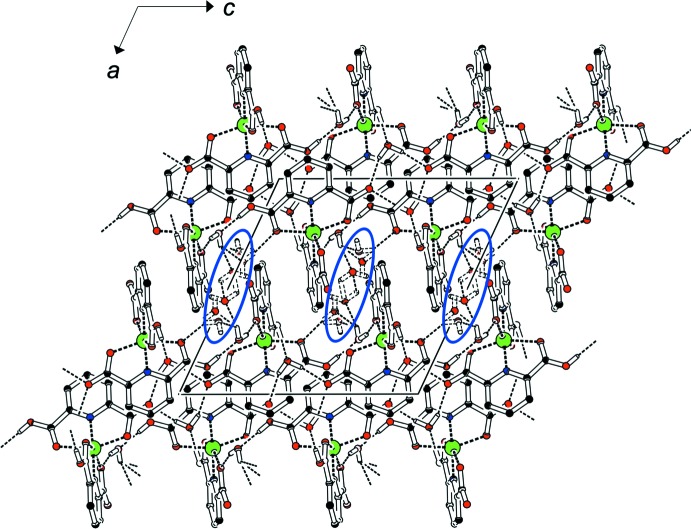
Hydrogen bonds in **2**; H atoms not involved in short contacts have been omitted. The disordered water mol­ecules highlighted in blue connect adjacent layers along [100].

In all compounds but **3**, classical O—H⋯O hydrogen bonds occur. In the isostructural solids **1** (Table 7 ▸) and **2** (Table 8 ▸) the well-ordered water mol­ecules associated with O9 and O10 connect complex mol­ecules *via* short hydroxyl-OH⋯water contacts and moderately strong H_2_O⋯carbonyl contacts into layers in the (100) plane. The disordered water mol­ecule associated with sites O11*A*, O11*B* and O11*C* links adjacent layers along [100] into a three-dimensional hydrogen-bonded network; Fig. 7 ▸ shows this arrangement for **2**.

Compound **4** is a two-dimensional coordination polymer extending parallel to (001); the hydroxyl group is involved both as donor and acceptor in the shortest hydrogen bonds (Table 9 ▸) within these layers. The longer hydrogen bonds subtended by the water mol­ecule O7 link successive layers in the third dimension along [001]. The Cl substituent in **5** accepts a rather long hydrogen bond from an aqua ligand of a neighbouring mol­ecule (Table 10 ▸); even without this inter­action, O—H⋯O contacts result in a three-dimensional hydrogen-bonded network. Table 11 ▸ compiles the hydrogen bonds in **6**. All classical hydrogen-bond donors find an acceptor in a suitable geometry, resulting in a three-dimensional network. In contrast to **4**, the hydroxyl group only acts as a hydrogen-bond donor.

## Conclusion and outlook   

In this article we compare coordination compounds of divalent Ni^II^ and Zn^II^ cations; they share similar ionic radii but differ with respect to their electron configuration. Crystal-field stabilization can be expected for the *d* ^8^ configuration of the former cation whereas no such effects will be observed for the latter with its fully occupied *d* subshell. A first very direct comparison can be made between compounds **1** and **2** with octa­hedral coordination of the metal cations, facilitated by their strict isotypism. In the octa­hedral environment, the *d* subshell of Ni^II^ splits into a set of three fully occupied *t*_2*g*_ and two half-occupied *e_g_* orbitals; the former induce a very regular coordination geometry. In contrast, the fully occupied and hence fully symmetric *d* subshell of Zn^II^ can adapt to any coordination mode. In line with this expectation the Ni^II^ complex **2** is significantly more regular than its Zn^II^ analogue **1**. Complexes **3** and **4** with only one fully deprotonated pyridine-2,6-di­carboxyl­ate ligand adopt a low-symmetry fivefold coordination with the Zn^II^ cation – very possible for a fully symmetric *d* ^10^ subshell without any preference for a certain ligand geometry. No such structures exist for the Ni^II^ complexes **5** and **6** of the same pyridine-2,6-di­carboxyl­ates: additional aqua ligands complete rather regular octa­hedral coordination environments about the crystal-field-stabilized transition-metal cation with its incomplete *d* subshell. Our analysis of structures with the same metal:ligand ratio, *i.e.* **1** *versus* **2** and **3**/**4** *versus* **5**/**6** consistently shows that the central Ni^II^ cations with their partially occupied *d* subshell induce the more regular, crystal-field-stabilized coordination geometry whereas the *d* ^10^-configured Zn^II^ cation can adapt to even very unsymmetrical coordination geometries. The structures reported here can be considered a direct experimental proof for the concept of crystal-field stabilization. With respect to our inter­est in extended structures (Kondraçka & Englert, 2008 ▸; Merkens & Englert, 2012 ▸; Merkens *et al.*, 2012 ▸; Kremer & Englert, 2018 ▸), we conclude that substituted pyridine-2,6-di­carboxyl­ates may well represent useful linkers between main-group cations in a 1:1 stoichiometry. In this case, the chelating and bridging coordination mode of the di­carboxyl­ato ligand induces a coordination sphere of low symmetry. We expect that the Ni^II^ complexes **5** and **6** are mononuclear because the additional aqua ligands allow the formation of a much more symmetric ligand field. Derivatives of crystal-field-stabilized transition-metal cations can probably not be isostructural with the coordination polymers **3** and **4**.

## Database survey   

Our database surveys aimed at complexes in which a metal is coordinated to the pyridine nitro­gen atoms and at least one carboxyl­ate oxygen of pyridine-2,6-di­carb­oxy­lic acid or one of its derivatives. They were conducted with Version 5.39 of the CSD (Groom *et al.*, 2016 ▸), including the updates of August 2018, and restricted to error-free entries without disorder for which atomic coordinates were available.

## Experimental   

### Synthesis and crystallization   

**Compound 1:**

Pyridine-2,6-di­carb­oxy­lic acid (H_2_Lig^1^) (247.5 mg, 1.48 mmol, Sigma–Aldrich) was dissolved in deionized water (11 ml) at 373 K. This solution was added to a solution of ZnCl_2_ (101.2 mg, 0.743 mmol) in deionized water (2 ml). Colourless rods were obtained after 15 minutes. Yield: 233.6 mg (0.517mmol, 69.8%). Analysis calculated for (**1**): ZnC_14_H_8_N_2_O_8_·3H_2_O): C 37.23, H 3.12, N 6.20; found: C 37.66, H 2.87, N 5.40. The thermal stability of **1** was investigated in detail; the result of the thermogravimetric analysis is represented in the supporting information to this article. It indicates that decomposition occurs in two steps: first, the three co-crystallized water mol­ecules are lost, followed by a second step probably associated with deca­rboxylation and slow concomitant decomposition.

**Compound 2:**

Pyridine-2,6-di­carb­oxy­lic acid (H_2_Lig^1^) (204.7 mg, 1.23 mmol, Sigma–Aldrich) was dissolved in deionized water (10 ml) at 373 K. This solution was added to a solution of NiCl_2_·6H_2_O (146.6 mg, 0.617 mmol) in deionized water (1 ml). Green rods were obtained after several days. Yield: 181.2 mg (0.407 mmol, 66.5%). Analysis calculated for **2**: NiC_14_H_8_N_2_O_8_×3(H_2_O): C 37.79, H 3.17, N 6.30; found: C 37.90, H 3.13, N 6.06.

**Compound 3:**

4-Chloro­pyridine-2,6-di­carb­oxy­lic acid (H_2_Lig^2^) (60 mg, 0.298 mmol, abcr) was dissolved in deionized water (10 ml) and heated to 368 K without stirring. ZnCl_2_ (60.2 mg, 0.442 mmol, Gruessing) was added to the solution. After 2 h, the heat source was removed, and the solution was left to cool to ambient temperature overnight. The crystals were obtained as colourless blocks. Yield: 128.0 mg (0.483 mmol, 64.9%). Analysis calculated for **3**: ZnC_7_H_2_ClNO_4_: C 31.73, H 0.76, N 5.29; found: C 31.66, H 0.93, N 5.19. Thermal analysis indicated stability of the compound up to a 670 K.

**Compound 4:**

4-Hy­droxy­pyridine-2,6-di­carb­oxy­lic acid (H_2_Lig^3^) (94.0 mg, 0.513 mmol, abcr) was dissolved in deionized water (11 ml) and heated to 368 K without stirring. ZnCl_2_ (210 mg, 1.54 mmol, Gruessing) was added to the solution. After 4 h, the heat source was removed, and the solution was left to cool to ambient temperature overnight. A large excess of metal salt was used to prevent the crystallization of the monohydrate of the ligand. The product was obtained as brown crystalline blocks. Yield: 67.2 mg (0.273 mmol, 53.1%). Although no visible decomposition was observed below 570 K, the analytical data indicate that the co-crystallized water mol­ecule evaporates upon drying of the crystals. Analysis calculated for **4** without H_2_O, ZnC_7_H_3_NO_5_: C 34.11, H 1.23, N 5.68; found: C 34.10, H 2.23, N 5.69.

**Compound 5:**

4-Chloro­pyridine-2,6-di­carb­oxy­lic acid (H_2_Lig^2^) (150 mg, 0.744 mmol, abcr) was dissolved in ethanol (10 ml). This solution was added to a solution of NiCl_2_·6H_2_O (176.9 mg, 0.744 mmol, Gruessing) in deionized water (4 ml). The crystals were obtained as green rods after several days. Yield: 101.3 mg (0.324 mmol, 43.6%). Despite the good match between the experimental and simulated powder patterns, no fully satisfactory microchemical analysis could be achieved. Analysis calculated for **5**: NiC_7_H_8_ClNO_7_: C 26.92, H 2.58, N 4.46; found: C 27.76, H 2.67, N 4.35. No visible decomposition was observed below 570 K.

In order to improve the match of the elemental analysis, an alternative synthesis was explored: 4-chloro­pyridine-2,6-di­carb­oxy­lic acid (H_2_Lig^2^) (94.6 mg, 0.469 mmol, abcr) and NiCO_3_ (115 mg, 0.469 mmol) were suspended in deionized water (8 ml). CO_2_ was evolved and the solids dissolved. The resulting solution was stored at 423 K for one h to evaporate most of the solvent and then kept at room temperature. Further evaporation over a period of one night lead to crystallization. Analysis calculated for **5**: NiC_7_H_8_ClNO_7_: C 26.92, H 2.58, N 4.46; found: C 26.00, H 2.98, N 4.39.

**Compound 6:**

4-Hy­droxy­pyridine-2,6-di­carb­oxy­lic acid (H_2_Lig^3^) (70.0 mg, 0.382 mmol, abcr) was dissolved in deionized water (11 ml) at 373 K. This solution was added to a solution of NiCl_2_·6H_2_O (181.7 mg, 0.764 mmol) in deionized water (1 ml). The product was obtained as brown crystalline blocks after several days. An excess of metal salt was used to prevent the crystallization of the monohydrate of the ligand. Yield: 75.8 mg (0.247 mmol, 64.6%). Analysis calculated for **6**: C_7_H_9_NNiO_8_·1.7(H_2_O): C 25.91, H 3.85, N 4.32; found: C 26.11, H 3.69, N 4.44. No visible decomposition was observed below 570 K.

For all solids **1**–**6** matching powder patterns (see supporting information) confirmed that the bulk samples essentially correspond to the structures derived from single crystal diffraction experiments.

### Powder diffraction   

X-ray powder diffraction experiments were performed at ambient temperature on flat samples with a Stoe STADI P diffractometer equipped with an image plate detector with constant *ω* angle of 55° using germanium–monochromated Cu *K*_α1_ radiation (λ = 1.54059 Å). Powder patterns for **1**–**6** are given in the supporting information.

### Refinement   

Crystal data, data collection parameters and convergence results for the single crystal X-ray diffraction experiments are summarized in Table 12 ▸. Non-hydrogen atoms were assigned anisotropic displacement parameters. H atoms attached to carbon were introduced into calculated positions and treated as riding with *U*_iso_(H) = 1.2*U*_eq_(C). H atoms attached to oxygen were located from difference-Fourier maps. In **1** and **2**, the coordinates of the hydrogen atoms in the carb­oxy­lic acid groups were refined and their *U*_iso_ values constrained to 1.5*U*_eq_(O); H atoms in water mol­ecules were refined as riding on O, in the geometry detected by the difference-Fourier syntheses with an idealized O—H distance of 0.84 Å. In **4**, the coordinates of the hy­droxy H atom were refined with an O—H distance restraint; H atoms in the water mol­ecules were refined as riding on O, in the geometry detected by the difference-Fourier syntheses with an idealized O—H distance of 0.84 Å. In **5**, the coordinates of the H atoms associated with the aqua ligands were refined with O—H distance restraints. In **6**, the coordinates of the H atoms associated with the aqua ligands and with hy­droxy group were refined with O—H distance restraints; H atoms attached to the co-crystallized water mol­ecules were refined as riding on oxygen, in the geometry detected by the difference-Fourier synthesis with an idealized O—-H distance of 0.84 Å. One of the solvent water mol­ecules in **1** and **2** is disordered over three mutually exclusive positions; the sum of its site occupancies was restrained to unity. In **5**, the non-coordinating carboxyl­ato O atom in the asymmetric unit was treated as disordered; the sum of its site occupancies was constrained to unity. In **6**, a water mol­ecule is in part located on a twofold axis, in part on a general position close to this axis. Tentative treatment of the electron density in this void with BYPASS/SQUEEZE (van der Sluis & Spek, 1990 ▸; Spek, 2009 ▸) suggests an overall content of 50 electrons, corresponding to five water mol­ecules per cell or 0.63 water mol­ecules per asymmetric unit, in good agreement with our refined water content of 0.7 mol­ecules per asymmetric unit. One of the two non-coordinating carboxyl­ato O atoms was treated as disordered over two positions; the sum of the site occupancies was constrained to unity. As the occupancies of the mutually exclusive sites converged to very different values, the minority site was only assigned an isotropic displacement parameter. Our structure model, with a disordered water mol­ecule in part located on a twofold axis and in part on a general position close to this axis, is similar to that of Fronczek (2015 ▸). In contrast, Cui *et al.* (2006 ▸) and Aghabozorg *et al.* (2007 ▸) have described the same water site by a single electron density maximum associated with a very large displacement parameter.

### Thermal analyses   

Thermogravimetric analyses (see supporting information) were performed under N_2_ with a heating rate of 5 K min ^−1^ for (1) and 10 K min ^−1^ for **3** with a Mettler Toledo TGA/SDTA 851e instrument.

## Supplementary Material

Crystal structure: contains datablock(s) 1, 2, 3, 4, 5, 6, global. DOI: 10.1107/S2056989019007461/wm5493sup1.cif

Structure factors: contains datablock(s) 1. DOI: 10.1107/S2056989019007461/wm54931sup2.hkl

Structure factors: contains datablock(s) 2. DOI: 10.1107/S2056989019007461/wm54932sup3.hkl

Structure factors: contains datablock(s) 3. DOI: 10.1107/S2056989019007461/wm54933sup4.hkl

Structure factors: contains datablock(s) 4. DOI: 10.1107/S2056989019007461/wm54934sup5.hkl

Structure factors: contains datablock(s) 5. DOI: 10.1107/S2056989019007461/wm54935sup6.hkl

Structure factors: contains datablock(s) 6. DOI: 10.1107/S2056989019007461/wm54936sup7.hkl

Thermal stability investigations and powder patterns. DOI: 10.1107/S2056989019007461/wm5493sup8.pdf

CCDC references: 1917869, 1917868, 1917867, 1917866, 1917865, 1917864

Additional supporting information:  crystallographic information; 3D view; checkCIF report

## Figures and Tables

**Table 1:** Selected geometric parameters (Å, °) for **1** [Chem scheme1]

Zn1—N2	2.0102 (15)	Zn1—O5	2.1245 (15)
Zn1—N1	2.0155 (15)	Zn1—O7	2.3093 (15)
Zn1—O1	2.0833 (14)	Zn1—O3	2.3312 (14)
			
N2—Zn1—N1	167.53 (6)	O1—Zn1—O7	94.21 (6)
N2—Zn1—O1	113.04 (6)	O5—Zn1—O7	152.42 (6)
N1—Zn1—O1	79.13 (6)	N2—Zn1—O3	94.15 (6)
N2—Zn1—O5	78.25 (6)	N1—Zn1—O3	73.57 (5)
N1—Zn1—O5	103.77 (6)	O1—Zn1—O3	152.62 (5)
O1—Zn1—O5	96.75 (6)	O5—Zn1—O3	91.74 (6)
N2—Zn1—O7	74.17 (6)	O7—Zn1—O3	90.01 (5)
N1—Zn1—O7	103.12 (5)		

**Table 2:** Selected geometric parameters (Å, °) for **2** [Chem scheme1]

Ni1—N1	1.9654 (15)	Ni1—O5	2.1036 (14)
Ni1—N2	1.9720 (16)	Ni1—O3	2.1666 (14)
Ni1—O1	2.0959 (14)	Ni1—O7	2.1940 (14)
			
N1—Ni1—N2	176.53 (6)	O1—Ni1—O3	156.09 (6)
N1—Ni1—O1	78.84 (6)	O5—Ni1—O3	92.85 (6)
N2—Ni1—O1	104.49 (6)	N1—Ni1—O7	104.37 (6)
N1—Ni1—O5	100.48 (6)	N2—Ni1—O7	76.60 (6)
N2—Ni1—O5	78.52 (6)	O1—Ni1—O7	93.18 (6)
O1—Ni1—O5	92.69 (6)	O5—Ni1—O7	155.12 (6)
N1—Ni1—O3	77.29 (6)	O3—Ni1—O7	91.51 (6)
N2—Ni1—O3	99.40 (6)		

**Table 3:** Selected geometric parameters (Å, °) for **3** [Chem scheme1]

Zn1—O2^i^	1.950 (2)	Zn1—O1	2.178 (2)
Zn1—O4^ii^	1.985 (2)	Zn1—O3	2.214 (2)
Zn1—N1	2.034 (2)		
			
O2^i^—Zn1—O4^ii^	101.71 (8)	N1—Zn1—O1	76.44 (9)
O2^i^—Zn1—N1	139.20 (10)	O2^i^—Zn1—O3	93.68 (9)
O4^ii^—Zn1—N1	117.79 (9)	O4^ii^—Zn1—O3	93.58 (9)
O2^i^—Zn1—O1	105.86 (9)	N1—Zn1—O3	75.04 (9)
O4^ii^—Zn1—O1	102.54 (9)	O1—Zn1—O3	151.30 (7)

Symmetry codes: (i) 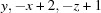; (ii) 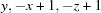.

**Table 4:** Selected geometric parameters (Å, °) for **4** [Chem scheme1]

Zn1—O2^i^	1.956 (3)	Zn1—O3	2.164 (3)
Zn1—O4^ii^	1.987 (3)	Zn1—O1	2.240 (3)
Zn1—N1	2.014 (3)		
			
O2^i^—Zn1—O4^ii^	102.10 (12)	N1—Zn1—O3	76.38 (11)
O2^i^—Zn1—N1	135.97 (12)	O2^i^—Zn1—O1	102.52 (12)
O4^ii^—Zn1—N1	121.41 (12)	O4^ii^—Zn1—O1	102.68 (12)
O2^i^—Zn1—O3	94.78 (12)	N1—Zn1—O1	75.85 (11)
O4^ii^—Zn1—O3	94.93 (12)	O3—Zn1—O1	151.96 (9)

Symmetry codes: (i) 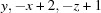; (ii) 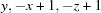.

**Table 5:** Selected geometric parameters (Å, °) for **5** [Chem scheme1]

Ni1—N1	1.975 (5)	Ni1—O3	2.036 (4)
Ni1—O4	2.023 (5)	Ni1—O1	2.131 (3)
Ni1—O3^i^	2.036 (4)	Ni1—O1^i^	2.131 (3)
			
N1—Ni1—O4	180.0	O3^i^—Ni1—O1	89.43 (14)
N1—Ni1—O3^i^	94.48 (9)	O3—Ni1—O1	92.48 (14)
O4—Ni1—O3^i^	85.52 (9)	N1—Ni1—O1^i^	77.70 (8)
N1—Ni1—O3	94.48 (9)	O4—Ni1—O1^i^	102.30 (8)
O4—Ni1—O3	85.52 (9)	O3^i^—Ni1—O1^i^	92.48 (14)
O3^i^—Ni1—O3	171.03 (19)	O3—Ni1—O1^i^	89.43 (14)
N1—Ni1—O1	77.70 (8)	O1—Ni1—O1^i^	155.40 (16)
O4—Ni1—O1	102.30 (8)		

Symmetry code: (i) 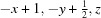.

**Table 6:** Selected geometric parameters (Å, °) for **6** [Chem scheme1]

Ni1—N1	1.9681 (17)	Ni1—O8	2.0848 (19)
Ni1—O7	2.0082 (17)	Ni1—O3	2.1205 (16)
Ni1—O6	2.0816 (18)	Ni1—O1	2.1833 (15)
			
N1—Ni1—O7	175.97 (7)	O6—Ni1—O3	93.58 (7)
N1—Ni1—O6	95.02 (7)	O8—Ni1—O3	91.91 (7)
O7—Ni1—O6	86.66 (7)	N1—Ni1—O1	76.84 (6)
N1—Ni1—O8	93.23 (7)	O7—Ni1—O1	106.90 (6)
O7—Ni1—O8	85.38 (8)	O6—Ni1—O1	88.60 (6)
O6—Ni1—O8	170.86 (7)	O8—Ni1—O1	89.44 (7)
N1—Ni1—O3	78.58 (7)	O3—Ni1—O1	155.42 (6)
O7—Ni1—O3	97.67 (6)		

**Table 7:** Hydrogen-bond geometry (Å, °) for **1** [Chem scheme1]

*D*—H⋯*A*	*D*—H	H⋯*A*	*D*⋯*A*	*D*—H⋯*A*
O4—H4*O*⋯O10^i^	0.86 (2)	1.60 (2)	2.451 (2)	173 (3)
O8—H8*O*⋯O9^ii^	0.86 (2)	1.62 (2)	2.478 (2)	170 (3)
O9—H9*O*⋯O2^iii^	0.84	1.94	2.752 (2)	164
O9—H9*P*⋯O2^i^	0.84	1.88	2.709 (2)	170
O10—H10*O*⋯O11*A* ^iv^	0.84	1.97	2.531 (8)	124
O10—H10*O*⋯O11*B* ^iv^	0.84	1.92	2.717 (6)	158
O10—H10*O*⋯O11*C* ^iv^	0.84	1.96	2.607 (7)	134
O10—H10*P*⋯O5	0.84	1.86	2.676 (2)	164
O11*A*—H11*P*⋯O3	0.85	2.11	2.875 (8)	149
O11*C*—H11*T*⋯O3	0.85	2.15	2.820 (8)	136

Symmetry codes: (i) 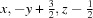; (ii) 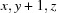; (iii) 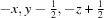; (iv) 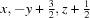.

**Table 8:** Hydrogen-bond geometry (Å, °) for **2** [Chem scheme1]

*D*—H⋯*A*	*D*—H	H⋯*A*	*D*⋯*A*	*D*—H⋯*A*
O4—H4*O*⋯O10^i^	0.84 (1)	1.61 (1)	2.456 (2)	177 (3)
O8—H8*O*⋯O9^ii^	0.84 (1)	1.63 (1)	2.462 (2)	171 (3)
O9—H9*O*⋯O2^iii^	0.84	1.94	2.751 (2)	162
O9—H9*P*⋯O2^i^	0.84	1.84	2.678 (2)	172
O10—H10*O*⋯O11*A* ^iv^	0.84	1.90	2.529 (5)	131
O10—H10*O*⋯O11*B* ^iv^	0.84	1.89	2.715 (8)	166
O10—H10*O*⋯O11*C* ^iv^	0.84	1.86	2.581 (7)	143
O10—H10*P*⋯O5	0.84	1.78	2.608 (2)	167
O11*A*—H11*P*⋯O3	0.85	2.08	2.842 (5)	149
O11*C*—H11*T*⋯O3	0.83	2.11	2.798 (8)	140

Symmetry codes: (i) 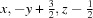; (ii) 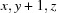; (iii) 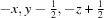; (iv) 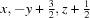.

**Table 9:** Hydrogen-bond geometry (Å, °) for **4** [Chem scheme1]

*D*—H⋯*A*	*D*—H	H⋯*A*	*D*⋯*A*	*D*—H⋯*A*
O5—H5*O*⋯O7	0.82 (3)	1.94 (3)	2.765 (4)	172 (6)
O6—H6*P*⋯O5^iii^	0.84	1.95	2.782 (4)	174
O7—H7*A*⋯O1^iv^	0.84	2.38	3.220 (4)	177
O7—H7*A*⋯O2^iv^	0.84	2.50	3.098 (3)	129
O7—H7*B*⋯O1^v^	0.84	2.38	3.220 (4)	177
O7—H7*B*⋯O2^v^	0.84	2.50	3.098 (3)	129

Symmetry codes: (iii) 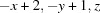; (iv) 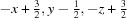; (v) 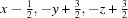.

**Table 10:** Hydrogen-bond geometry (Å, °) for **5** [Chem scheme1]

*D*—H⋯*A*	*D*—H	H⋯*A*	*D*⋯*A*	*D*—H⋯*A*
O3—H3*P*⋯O2*B* ^ii^	0.81 (2)	2.10 (3)	2.90 (3)	174 (5)
O3—H3*P*⋯O2*A* ^ii^	0.81 (2)	1.85 (3)	2.628 (18)	162 (6)
O3—H3*O*⋯Cl1^iii^	0.79 (2)	2.75 (3)	3.464 (4)	150 (5)
O4—H4*O*⋯O2*B* ^iv^	0.79 (2)	2.53 (6)	2.95 (3)	115 (5)
O4—H4*O*⋯O2*B* ^v^	0.79 (2)	2.11 (5)	2.83 (3)	151 (6)

Symmetry codes: (ii) 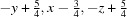; (iii) 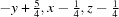; (iv) 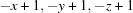; (v) 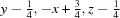.

**Table 11:** Hydrogen-bond geometry (Å, °) for **6** [Chem scheme1]

*D*—H⋯*A*	*D*—H	H⋯*A*	*D*⋯*A*	*D*—H⋯*A*
O5—H5*O*⋯O1^i^	0.84	1.79	2.624 (2)	169
O6—H6*O*⋯O2^ii^	0.81 (2)	2.07 (2)	2.836 (2)	158 (4)
O6—H6*O*⋯O5^iii^	0.81 (2)	2.66 (4)	3.130 (2)	119 (3)
O6—H6*P*⋯O9^iv^	0.84 (2)	2.00 (2)	2.821 (3)	167 (4)
O7—H7*P*⋯O3^v^	0.81 (2)	1.96 (2)	2.751 (2)	166 (4)
O7—H7*O*⋯O10	0.82 (2)	2.24 (3)	2.989 (5)	152 (4)
O7—H7*O*⋯O11	0.82 (2)	1.77 (2)	2.574 (5)	170 (5)
O8—H8*P*⋯O9^v^	0.81 (2)	2.00 (2)	2.811 (3)	173 (4)
O8—H8*O*⋯O2^vi^	0.82 (2)	1.88 (2)	2.691 (2)	170 (3)
O9—H9*O*⋯O4*A* ^iv^	0.84	2.12	2.934 (3)	161
O9—H9*O*⋯O4*B* ^iv^	0.84	2.67	3.51 (2)	175
O9—H9*P*⋯O4*A*	0.84	1.93	2.729 (3)	159
O9—H9*P*⋯O4*B*	0.84	1.98	2.693 (17)	142

Symmetry codes: (i) 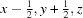; (ii) 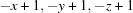; (iii) 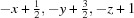; (iv) 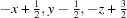; (v) 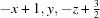; (vi) 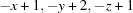.

**Table:** 

	**1**	**2**	**3**
Crystal data			
Chemical formula	[Zn(C_7_H_4_NO_4_)_2_]·3H_2_O	[Ni(C_7_H_4_NO_4_)_2_]·3H_2_O	[Zn(C_7_H_2_ClNO_4_)]
*M*_r_	451.64	444.98	264.94
Crystal system, space group	Monoclinic, *P*2_1_/*c*	Monoclinic, *P*2_1_/*c*	Tetragonal, *P* 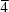 2_1_ *c*
Temperature (K)	220	250	100
*a*, *b*, *c* (Å)	13.9953 (8), 10.0081 (6), 13.7330 (8)	13.6651 (15), 10.0207 (11), 13.7696 (15)	10.0293 (5), 10.0293 (5), 16.8924 (9)
α, β, γ (°)	90, 116.4303 (14), 90	90, 115.109 (2), 90	90, 90, 90
*V* (Å^3^)	1722.48 (18)	1707.3 (3)	1699.15 (19)
*Z*	4	4	8
Radiation type	Mo *K*α	Mo *K*α	Mo *K*α
μ (mm^−1^)	1.49	1.20	3.19
Crystal size (mm)	0.28 × 0.18 × 0.18	0.40 × 0.12 × 0.12	0.25 × 0.10 × 0.10

Data collection			
Diffractometer	Bruker APEX CCD	Bruker APEX CCD	Bruker APEX CCD
Absorption correction	Multi-scan *SADABS* (Bruker, 2008 ▸)	Multi-scan *SADABS* (Bruker, 2008 ▸)	Multi-scan *SADABS* (Bruker, 2008 ▸)
*T*_min_, *T*_max_	0.877, 1.000	0.821, 1.000	0.590, 0.746
No. of measured, independent and observed [*I* > 2σ(*I*)] reflections	56614, 5777, 4180	25725, 5107, 3629	25321, 2592, 2266
*R*_int_	0.054	0.043	0.059
(sin θ/λ)_max_ (Å^−1^)	0.737	0.718	0.717

Refinement			
*R*[*F* ^2^ > 2σ(*F* ^2^)], *wR*(*F* ^2^), *S*	0.037, 0.105, 1.04	0.039, 0.128, 1.03	0.026, 0.064, 1.07
No. of reflections	5777	5107	2592
No. of parameters	263	263	128
No. of restraints	2	2	0
H-atom treatment	H atoms treated by a mixture of independent and constrained refinement	H atoms treated by a mixture of independent and constrained refinement	H-atom parameters constrained
Δρ_max_, Δρ_min_ (e Å^−3^)	0.56, −0.35	0.56, −0.35	0.42, −0.31
Absolute structure	–	–	Refined as an inversion twin
Absolute structure parameter	–	–	0.41 (2)

**Table:** 

	**4**	**5**	**6**
Crystal data			
Chemical formula	[Zn(C_7_H_3_NO_5_)]·H_2_O	[Ni(C_7_H_2_ClNO_4_)(H_2_O)_3_]	[Ni(C_7_H_3_NO_5_)(H_2_O)_3_]·1.7H_2_O
*M*_r_	529.02	312.30	324.49
Crystal system, space group	Tetragonal, *P* 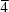 2_1_ *c*	Tetragonal, *I*4_1_/*a*	Monoclinic, *C*2/*c*
Temperature (K)	100	250	100
*a*, *b*, *c* (Å)	10.050 (1), 10.050 (1), 16.5060 (16)	9.544 (2), 9.544 (2), 23.361 (5)	14.7249 (11), 6.8538 (5), 22.3510 (16)
α, β, γ (°)	90, 90, 90	90, 90, 90	90, 90.355 (1), 90
*V* (Å^3^)	1667.1 (4)	2127.9 (11)	2255.7 (3)
*Z*	4	8	8
Radiation type	Mo *K*α	Mo *K*α	Mo *K*α
μ (mm^−1^)	2.96	2.10	1.77
Crystal size (mm)	0.16 × 0.10 × 0.09	0.40 × 0.25 × 0.18	0.33 × 0.32 × 0.12

Data collection			
Diffractometer	Bruker APEX CCD	Bruker APEX CCD	Bruker APEX CCD
Absorption correction	Multi-scan *SADABS* (Bruker, 2008 ▸)	Multi-scan *SADABS* (Bruker, 2008 ▸)	Multi-scan *SADABS* (Bruker, 2008 ▸)
*T*_min_, *T*_max_	0.497, 0.746	0.468, 0.745	0.569, 0.746
No. of measured, independent and observed [*I* > 2σ(*I*)] reflections	24393, 2567, 2371	12540, 1127, 935	16693, 3368, 2963
*R*_int_	0.047	0.092	0.027
(sin θ/λ)_max_ (Å^−1^)	0.723	0.634	0.725

Refinement			
*R*[*F* ^2^ > 2σ(*F* ^2^)], *wR*(*F* ^2^), *S*	0.033, 0.079, 1.07	0.050, 0.127, 1.14	0.039, 0.106, 1.17
No. of reflections	2567	1127	3368
No. of parameters	141	99	214
No. of restraints	1	8	12
H-atom treatment	H atoms treated by a mixture of independent and constrained refinement	H atoms treated by a mixture of independent and constrained refinement	H atoms treated by a mixture of independent and constrained refinement
Δρ_max_, Δρ_min_ (e Å^−3^)	0.70, −0.46	0.50, −0.51	0.79, −0.52
Absolute structure	Refined as an inversion twin	?	–
Absolute structure parameter	0.47 (2)	?	–

Computer programs: *SMART* (Bruker, 2001 ▸), *SAINT-Plus* (Bruker, 2009 ▸), *SHELXT* (Sheldrick, 2015*a* ▸), *SHELXL2013* (Sheldrick, 2015*b* ▸) and *Mercury* (Macrae *et al.* (2006 ▸).

## supplementary crystallographic information

### Bis(6-carboxypicolinato)zinc(II) trihydrate (1) Crystal data

**Table:**

[Zn(C_7_H_4_NO_4_)_2_]·3H_2_O	*F*(000) = 920
*M**_r_* = 451.64	*D*_x_ = 1.742 Mg m^−^^3^
Monoclinic, *P*2_1_/*c*	Mo *K*α radiation, λ = 0.71073 Å
*a* = 13.9953 (8) Å	Cell parameters from 7944 reflections
*b* = 10.0081 (6) Å	θ = 2.6–23.8°
*c* = 13.7330 (8) Å	µ = 1.49 mm^−^^1^
β = 116.4303 (14)°	*T* = 220 K
*V* = 1722.48 (18) Å^3^	Rod, colourless
*Z* = 4	0.28 × 0.18 × 0.18 mm

### Bis(6-carboxypicolinato)zinc(II) trihydrate (1) Data collection

**Table:**

Bruker APEX CCD diffractometer	4180 reflections with *I* > 2σ(*I*)
Radiation source: microsource	*R*_int_ = 0.054
ω scans	θ_max_ = 31.6°, θ_min_ = 1.6°
Absorption correction: multi-scan SADABS (Bruker, 2008)	*h* = −20→20
*T*_min_ = 0.877, *T*_max_ = 1.000	*k* = −14→14
56614 measured reflections	*l* = −20→20
5777 independent reflections	

### Bis(6-carboxypicolinato)zinc(II) trihydrate (1) Refinement

**Table:**

Refinement on *F*^2^	2 restraints
Least-squares matrix: full	Hydrogen site location: mixed
*R*[*F*^2^ > 2σ(*F*^2^)] = 0.037	H atoms treated by a mixture of independent and constrained refinement
*wR*(*F*^2^) = 0.105	*w* = 1/[σ^2^(*F*_o_^2^) + (0.0416*P*)^2^ + 1.0164*P*] where *P* = (*F*_o_^2^ + 2*F*_c_^2^)/3
*S* = 1.04	(Δ/σ)_max_ = 0.001
5777 reflections	Δρ_max_ = 0.56 e Å^−^^3^
263 parameters	Δρ_min_ = −0.35 e Å^−^^3^

### Bis(6-carboxypicolinato)zinc(II) trihydrate (1) Special details

**Table:**

Geometry. All esds (except the esd in the dihedral angle between two l.s. planes) are estimated using the full covariance matrix. The cell esds are taken into account individually in the estimation of esds in distances, angles and torsion angles; correlations between esds in cell parameters are only used when they are defined by crystal symmetry. An approximate (isotropic) treatment of cell esds is used for estimating esds involving l.s. planes.

### Bis(6-carboxypicolinato)zinc(II) trihydrate (1) Fractional atomic coordinates and isotropic or equivalent isotropic displacement parameters (Å^2^)

**Table:**

	*x*	*y*	*z*	*U*_iso_*/*U*_eq_	Occ. (<1)
Zn1	0.25020 (2)	0.99549 (2)	0.26138 (2)	0.03318 (8)	
O1	0.18718 (11)	1.00856 (15)	0.37285 (11)	0.0362 (3)	
O2	0.03924 (12)	0.96743 (16)	0.39055 (12)	0.0394 (3)	
O3	0.23801 (11)	0.94497 (16)	0.09052 (11)	0.0376 (3)	
O4	0.12629 (14)	0.84401 (16)	−0.06458 (12)	0.0423 (3)	
H4O	0.174 (2)	0.854 (3)	−0.088 (2)	0.063*	
O5	0.34985 (13)	0.82750 (15)	0.33256 (13)	0.0429 (3)	
O6	0.52279 (16)	0.7743 (2)	0.42790 (16)	0.0655 (5)	
O7	0.22321 (11)	1.21877 (14)	0.21460 (12)	0.0368 (3)	
O8	0.31855 (13)	1.40197 (16)	0.22226 (14)	0.0445 (4)	
H8O	0.2576 (17)	1.437 (3)	0.180 (2)	0.067*	
N1	0.10463 (12)	0.91469 (15)	0.17479 (12)	0.0273 (3)	
N2	0.39644 (11)	1.07655 (17)	0.31645 (12)	0.0296 (3)	
C1	0.04345 (14)	0.90384 (18)	0.22605 (14)	0.0286 (3)	
C2	−0.05478 (15)	0.8403 (2)	0.17755 (16)	0.0351 (4)	
H2	−0.097679	0.832445	0.213942	0.042*	
C3	−0.08801 (16)	0.7885 (2)	0.07356 (17)	0.0379 (4)	
H3	−0.154381	0.745240	0.038598	0.045*	
C4	−0.02338 (16)	0.80052 (19)	0.02125 (16)	0.0341 (4)	
H4	−0.044877	0.765742	−0.048981	0.041*	
C5	0.07331 (14)	0.86496 (18)	0.07525 (14)	0.0285 (3)	
C6	0.09289 (15)	0.96503 (19)	0.33896 (15)	0.0307 (4)	
C7	0.15363 (16)	0.88719 (19)	0.03184 (15)	0.0316 (4)	
C8	0.47935 (15)	0.9954 (2)	0.36847 (15)	0.0342 (4)	
C9	0.58359 (16)	1.0440 (3)	0.40976 (17)	0.0457 (5)	
H9	0.642028	0.987024	0.447096	0.055*	
C10	0.59927 (17)	1.1759 (3)	0.39499 (19)	0.0489 (6)	
H10	0.668946	1.210052	0.421825	0.059*	
C11	0.51240 (16)	1.2593 (3)	0.34041 (17)	0.0424 (5)	
H11	0.521956	1.350086	0.329531	0.051*	
C12	0.41133 (14)	1.2049 (2)	0.30250 (15)	0.0323 (4)	
C13	0.45005 (18)	0.8530 (2)	0.37782 (17)	0.0418 (5)	
C14	0.30825 (15)	1.2785 (2)	0.24167 (15)	0.0323 (4)	
O9	0.15287 (13)	0.52698 (17)	0.10985 (13)	0.0443 (4)	
H9O	0.100266	0.513987	0.122694	0.053*	
H9P	0.124646	0.529706	0.041594	0.053*	
O10	0.25352 (15)	0.61084 (17)	0.36169 (14)	0.0536 (4)	
H10O	0.309944	0.565544	0.386735	0.064*	
H10P	0.275684	0.677054	0.340055	0.064*	
O11A	0.3323 (7)	1.0949 (9)	−0.0231 (7)	0.0653 (12)*	0.283 (5)
H11O	0.283003	1.152053	−0.052998	0.078*	0.283 (5)
H11P	0.327964	1.056784	0.029852	0.078*	0.283 (5)
O11B	0.4329 (5)	1.0244 (6)	−0.0055 (5)	0.0653 (12)*	0.371 (5)
H11Q	0.452158	0.999978	0.059700	0.078*	0.371 (5)
H11R	0.470448	1.022909	−0.039770	0.078*	0.371 (5)
O11C	0.3815 (7)	1.0466 (8)	0.0158 (6)	0.0653 (12)*	0.321 (6)
H11S	0.443940	1.014077	0.041684	0.078*	0.321 (6)
H11T	0.341329	1.061387	0.046024	0.078*	0.321 (6)

### Bis(6-carboxypicolinato)zinc(II) trihydrate (1) Atomic displacement parameters (Å^2^)

**Table:**

	*U*^11^	*U*^22^	*U*^33^	*U*^12^	*U*^13^	*U*^23^
Zn1	0.02530 (11)	0.03862 (14)	0.03201 (12)	−0.00359 (8)	0.00950 (9)	−0.00199 (9)
O1	0.0296 (6)	0.0479 (8)	0.0298 (7)	−0.0058 (6)	0.0121 (5)	−0.0074 (6)
O2	0.0382 (7)	0.0507 (9)	0.0347 (7)	−0.0027 (6)	0.0211 (6)	−0.0046 (6)
O3	0.0357 (7)	0.0452 (8)	0.0334 (7)	−0.0045 (6)	0.0168 (6)	−0.0053 (6)
O4	0.0551 (9)	0.0451 (8)	0.0305 (7)	−0.0081 (7)	0.0225 (7)	−0.0081 (6)
O5	0.0475 (9)	0.0386 (8)	0.0426 (8)	0.0067 (6)	0.0201 (7)	0.0061 (6)
O6	0.0645 (12)	0.0670 (12)	0.0634 (12)	0.0364 (10)	0.0270 (10)	0.0235 (10)
O7	0.0258 (6)	0.0377 (8)	0.0393 (7)	−0.0005 (5)	0.0077 (5)	0.0012 (6)
O8	0.0408 (8)	0.0393 (8)	0.0520 (9)	−0.0021 (7)	0.0193 (7)	0.0085 (7)
N1	0.0272 (7)	0.0273 (7)	0.0254 (7)	0.0001 (6)	0.0099 (6)	−0.0002 (5)
N2	0.0224 (6)	0.0407 (9)	0.0241 (7)	0.0030 (6)	0.0088 (5)	0.0005 (6)
C1	0.0287 (8)	0.0280 (8)	0.0281 (8)	0.0013 (7)	0.0118 (7)	0.0021 (6)
C2	0.0326 (9)	0.0347 (10)	0.0376 (10)	−0.0048 (7)	0.0153 (8)	−0.0001 (8)
C3	0.0317 (9)	0.0360 (10)	0.0384 (10)	−0.0087 (8)	0.0088 (8)	−0.0029 (8)
C4	0.0370 (10)	0.0293 (9)	0.0285 (9)	−0.0026 (7)	0.0078 (7)	−0.0027 (7)
C5	0.0320 (9)	0.0250 (8)	0.0249 (8)	0.0013 (7)	0.0094 (7)	0.0013 (6)
C6	0.0315 (9)	0.0309 (9)	0.0287 (8)	0.0017 (7)	0.0124 (7)	0.0012 (7)
C7	0.0387 (10)	0.0276 (9)	0.0286 (8)	0.0029 (7)	0.0150 (7)	0.0011 (7)
C8	0.0276 (8)	0.0514 (12)	0.0232 (8)	0.0090 (8)	0.0109 (7)	0.0018 (8)
C9	0.0254 (9)	0.0793 (17)	0.0296 (9)	0.0107 (10)	0.0097 (7)	−0.0009 (10)
C10	0.0255 (9)	0.0786 (18)	0.0403 (11)	−0.0070 (10)	0.0125 (8)	−0.0045 (11)
C11	0.0325 (10)	0.0581 (14)	0.0362 (10)	−0.0121 (9)	0.0149 (8)	−0.0045 (9)
C12	0.0281 (8)	0.0428 (10)	0.0250 (8)	−0.0030 (7)	0.0110 (7)	−0.0020 (7)
C13	0.0437 (11)	0.0502 (12)	0.0328 (10)	0.0182 (9)	0.0184 (9)	0.0072 (9)
C14	0.0316 (9)	0.0384 (10)	0.0261 (8)	−0.0018 (7)	0.0122 (7)	−0.0009 (7)
O9	0.0416 (8)	0.0559 (10)	0.0343 (7)	0.0058 (7)	0.0159 (7)	0.0084 (7)
O10	0.0737 (12)	0.0474 (10)	0.0563 (10)	0.0038 (8)	0.0438 (9)	0.0023 (8)

### Bis(6-carboxypicolinato)zinc(II) trihydrate (1) Geometric parameters (Å, º)

**Table:**

Zn1—N2	2.0102 (15)	C3—C4	1.388 (3)
Zn1—N1	2.0155 (15)	C3—H3	0.9400
Zn1—O1	2.0833 (14)	C4—C5	1.380 (3)
Zn1—O5	2.1245 (15)	C4—H4	0.9400
Zn1—O7	2.3093 (15)	C5—C7	1.505 (3)
Zn1—O3	2.3312 (14)	C8—C9	1.397 (3)
O1—C6	1.265 (2)	C8—C13	1.504 (3)
O2—C6	1.241 (2)	C9—C10	1.368 (4)
O3—C7	1.237 (2)	C9—H9	0.9400
O4—C7	1.279 (2)	C10—C11	1.388 (3)
O4—H4O	0.856 (17)	C10—H10	0.9400
O5—C13	1.281 (3)	C11—C12	1.383 (3)
O6—C13	1.228 (3)	C11—H11	0.9400
O7—C14	1.232 (2)	C12—C14	1.500 (3)
O8—C14	1.286 (2)	O9—H9O	0.8400
O8—H8O	0.862 (17)	O9—H9P	0.8401
N1—C5	1.332 (2)	O10—H10O	0.8400
N1—C1	1.333 (2)	O10—H10P	0.8400
N2—C12	1.328 (3)	O11A—H11O	0.8482
N2—C8	1.334 (2)	O11A—H11P	0.8477
C1—C2	1.387 (3)	O11B—H11Q	0.8485
C1—C6	1.518 (3)	O11B—H11R	0.8473
C2—C3	1.390 (3)	O11C—H11S	0.8482
C2—H2	0.9400	O11C—H11T	0.8472
			
N2—Zn1—N1	167.53 (6)	C5—C4—H4	120.9
N2—Zn1—O1	113.04 (6)	C3—C4—H4	120.9
N1—Zn1—O1	79.13 (6)	N1—C5—C4	121.48 (17)
N2—Zn1—O5	78.25 (6)	N1—C5—C7	112.87 (16)
N1—Zn1—O5	103.77 (6)	C4—C5—C7	125.65 (17)
O1—Zn1—O5	96.75 (6)	O2—C6—O1	125.48 (18)
N2—Zn1—O7	74.17 (6)	O2—C6—C1	118.34 (17)
N1—Zn1—O7	103.12 (5)	O1—C6—C1	116.18 (16)
O1—Zn1—O7	94.21 (6)	O3—C7—O4	126.24 (19)
O5—Zn1—O7	152.42 (6)	O3—C7—C5	118.40 (16)
N2—Zn1—O3	94.15 (6)	O4—C7—C5	115.35 (17)
N1—Zn1—O3	73.57 (5)	N2—C8—C9	120.5 (2)
O1—Zn1—O3	152.62 (5)	N2—C8—C13	114.68 (17)
O5—Zn1—O3	91.74 (6)	C9—C8—C13	124.81 (19)
O7—Zn1—O3	90.01 (5)	C10—C9—C8	118.9 (2)
C6—O1—Zn1	114.93 (12)	C10—C9—H9	120.5
C7—O3—Zn1	112.20 (12)	C8—C9—H9	120.5
C7—O4—H4O	115 (2)	C9—C10—C11	120.0 (2)
C13—O5—Zn1	114.70 (14)	C9—C10—H10	120.0
C14—O7—Zn1	111.66 (13)	C11—C10—H10	120.0
C14—O8—H8O	111 (2)	C12—C11—C10	118.0 (2)
C5—N1—C1	121.07 (16)	C12—C11—H11	121.0
C5—N1—Zn1	122.67 (12)	C10—C11—H11	121.0
C1—N1—Zn1	116.06 (12)	N2—C12—C11	121.75 (19)
C12—N2—C8	120.74 (17)	N2—C12—C14	112.43 (16)
C12—N2—Zn1	122.32 (12)	C11—C12—C14	125.8 (2)
C8—N2—Zn1	116.94 (14)	O6—C13—O5	126.8 (2)
N1—C1—C2	121.08 (17)	O6—C13—C8	117.8 (2)
N1—C1—C6	113.51 (15)	O5—C13—C8	115.38 (17)
C2—C1—C6	125.41 (17)	O7—C14—O8	125.88 (18)
C1—C2—C3	118.11 (18)	O7—C14—C12	119.36 (18)
C1—C2—H2	120.9	O8—C14—C12	114.76 (17)
C3—C2—H2	120.9	H9O—O9—H9P	102.6
C4—C3—C2	120.13 (18)	H10O—O10—H10P	98.2
C4—C3—H3	119.9	H11O—O11A—H11P	111.4
C2—C3—H3	119.9	H11Q—O11B—H11R	126.6
C5—C4—C3	118.13 (18)	H11S—O11C—H11T	130.3

### Bis(6-carboxypicolinato)zinc(II) trihydrate (1) Hydrogen-bond geometry (Å, º)

**Table:**

*D*—H···*A*	*D*—H	H···*A*	*D*···*A*	*D*—H···*A*
O4—H4*O*···O10^i^	0.86 (2)	1.60 (2)	2.451 (2)	173 (3)
O8—H8*O*···O9^ii^	0.86 (2)	1.62 (2)	2.478 (2)	170 (3)
C2—H2···O7^iii^	0.94	2.62	3.512 (2)	158
C4—H4···O7^iv^	0.94	2.52	3.207 (2)	130
C9—H9···O1^v^	0.94	2.56	3.310 (3)	137
O9—H9*O*···O2^iii^	0.84	1.94	2.752 (2)	164
O9—H9*P*···O2^i^	0.84	1.88	2.709 (2)	170
O10—H10*O*···O11*A*^vi^	0.84	1.97	2.531 (8)	124
O10—H10*O*···O11*B*^vi^	0.84	1.92	2.717 (6)	158
O10—H10*O*···O11*C*^vi^	0.84	1.96	2.607 (7)	134
O10—H10*P*···O5	0.84	1.86	2.676 (2)	164
O11*A*—H11*P*···O3	0.85	2.11	2.875 (8)	149
O11*C*—H11*T*···O3	0.85	2.15	2.820 (8)	136

Symmetry codes: (i) *x*, −*y*+3/2, *z*−1/2; (ii) *x*, *y*+1, *z*; (iii) −*x*, *y*−1/2, −*z*+1/2; (iv) −*x*, −*y*+2, −*z*; (v) −*x*+1, −*y*+2, −*z*+1; (vi) *x*, −*y*+3/2, *z*+1/2.

### Bis(6-carboxypicolinato)nickel(II) trihydrate (2) Crystal data

**Table:**

[Ni(C_7_H_4_NO_4_)_2_]·3H_2_O	*F*(000) = 912
*M**_r_* = 444.98	*D*_x_ = 1.731 Mg m^−^^3^
Monoclinic, *P*2_1_/*c*	Mo *K*α radiation, λ = 0.71073 Å
*a* = 13.6651 (15) Å	Cell parameters from 3808 reflections
*b* = 10.0207 (11) Å	θ = 2.6–24.0°
*c* = 13.7696 (15) Å	µ = 1.20 mm^−^^1^
β = 115.109 (2)°	*T* = 250 K
*V* = 1707.3 (3) Å^3^	Rod, green
*Z* = 4	0.40 × 0.12 × 0.12 mm

### Bis(6-carboxypicolinato)nickel(II) trihydrate (2) Data collection

**Table:**

Bruker APEX CCD diffractometer	5107 independent reflections
Radiation source: microsource	3629 reflections with *I* > 2σ(*I*)
Multilayer optics monochromator	*R*_int_ = 0.043
ω scans	θ_max_ = 30.7°, θ_min_ = 1.7°
Absorption correction: multi-scan SADABS (Bruker, 2008)	*h* = −19→19
*T*_min_ = 0.821, *T*_max_ = 1.000	*k* = −14→14
25725 measured reflections	*l* = −18→18

### Bis(6-carboxypicolinato)nickel(II) trihydrate (2) Refinement

**Table:**

Refinement on *F*^2^	2 restraints
Least-squares matrix: full	Hydrogen site location: mixed
*R*[*F*^2^ > 2σ(*F*^2^)] = 0.039	H atoms treated by a mixture of independent and constrained refinement
*wR*(*F*^2^) = 0.128	*w* = 1/[σ^2^(*F*_o_^2^) + (0.0751*P*)^2^] where *P* = (*F*_o_^2^ + 2*F*_c_^2^)/3
*S* = 1.03	(Δ/σ)_max_ = 0.001
5107 reflections	Δρ_max_ = 0.56 e Å^−^^3^
263 parameters	Δρ_min_ = −0.35 e Å^−^^3^

### Bis(6-carboxypicolinato)nickel(II) trihydrate (2) Special details

**Table:**

Geometry. All esds (except the esd in the dihedral angle between two l.s. planes) are estimated using the full covariance matrix. The cell esds are taken into account individually in the estimation of esds in distances, angles and torsion angles; correlations between esds in cell parameters are only used when they are defined by crystal symmetry. An approximate (isotropic) treatment of cell esds is used for estimating esds involving l.s. planes.

### Bis(6-carboxypicolinato)nickel(II) trihydrate (2) Fractional atomic coordinates and isotropic or equivalent isotropic displacement parameters (Å^2^)

**Table:**

	*x*	*y*	*z*	*U*_iso_*/*U*_eq_	Occ. (<1)
Ni1	0.24979 (2)	0.99449 (2)	0.24731 (2)	0.02691 (10)	
O1	0.19331 (12)	1.01090 (13)	0.36653 (12)	0.0338 (3)	
O2	0.04726 (12)	0.97036 (16)	0.39563 (12)	0.0383 (3)	
O3	0.24067 (11)	0.94554 (15)	0.09043 (11)	0.0348 (3)	
O4	0.12899 (14)	0.84593 (16)	−0.06334 (12)	0.0428 (4)	
H4O	0.1726 (19)	0.858 (3)	−0.091 (2)	0.064*	
O5	0.34228 (12)	0.82400 (14)	0.31849 (12)	0.0390 (3)	
O6	0.51339 (15)	0.7616 (2)	0.41861 (16)	0.0651 (5)	
O7	0.22425 (11)	1.20706 (14)	0.20609 (12)	0.0345 (3)	
O8	0.31904 (12)	1.39512 (15)	0.22348 (13)	0.0426 (4)	
H8O	0.2599 (12)	1.429 (3)	0.1812 (18)	0.064*	
O9	0.15328 (14)	0.51674 (16)	0.10952 (13)	0.0431 (4)	
H9O	0.099413	0.503432	0.122688	0.052*	
H9P	0.125443	0.519432	0.042198	0.052*	
O10	0.25374 (15)	0.61017 (16)	0.35421 (13)	0.0513 (4)	
H10O	0.310150	0.564961	0.386267	0.062*	
H10P	0.276030	0.676781	0.332447	0.062*	
O11A	0.3367 (4)	1.0882 (5)	−0.0253 (4)	0.0577 (11)*	0.400 (5)
H11O	0.287358	1.145334	−0.055164	0.069*	0.400 (5)
H11P	0.332319	1.050065	0.027686	0.069*	0.400 (5)
O11B	0.4335 (7)	1.0224 (7)	−0.0113 (7)	0.0577 (11)*	0.251 (5)
H11Q	0.452844	0.998035	0.053904	0.069*	0.251 (5)
H11R	0.471134	1.020965	−0.045566	0.069*	0.251 (5)
O11C	0.3853 (7)	1.0352 (7)	0.0095 (6)	0.0577 (11)*	0.316 (5)
H11S	0.447772	1.002625	0.035351	0.069*	0.316 (5)
H11T	0.345162	1.049934	0.039691	0.069*	0.316 (5)
N1	0.10530 (12)	0.91467 (15)	0.17465 (12)	0.0259 (3)	
N2	0.39703 (12)	1.06957 (17)	0.31357 (12)	0.0282 (3)	
C1	0.04515 (15)	0.90427 (18)	0.22897 (15)	0.0273 (4)	
C2	−0.05419 (16)	0.8408 (2)	0.18435 (17)	0.0349 (4)	
H2	−0.096504	0.832386	0.222898	0.042*	
C3	−0.08993 (17)	0.7894 (2)	0.08062 (17)	0.0363 (5)	
H3	−0.157642	0.747267	0.048053	0.044*	
C4	−0.02563 (16)	0.80065 (19)	0.02566 (16)	0.0330 (4)	
H4	−0.048272	0.765639	−0.043847	0.040*	
C5	0.07249 (15)	0.86464 (18)	0.07594 (15)	0.0274 (4)	
C6	0.09866 (16)	0.96665 (19)	0.33975 (15)	0.0284 (4)	
C7	0.15467 (16)	0.88739 (18)	0.03159 (15)	0.0300 (4)	
C8	0.47876 (17)	0.9870 (2)	0.36699 (16)	0.0327 (5)	
C9	0.58453 (17)	1.0347 (3)	0.41499 (17)	0.0422 (5)	
H9	0.642284	0.976971	0.453447	0.051*	
C10	0.60270 (18)	1.1673 (3)	0.40509 (19)	0.0464 (6)	
H10	0.673571	1.201014	0.436732	0.056*	
C11	0.51676 (17)	1.2525 (2)	0.34838 (17)	0.0404 (5)	
H11	0.528277	1.343723	0.340975	0.049*	
C12	0.41416 (15)	1.1987 (2)	0.30345 (15)	0.0300 (4)	
C13	0.44463 (18)	0.8449 (2)	0.36941 (18)	0.0396 (5)	
C14	0.31013 (16)	1.2704 (2)	0.23926 (15)	0.0308 (4)	

### Bis(6-carboxypicolinato)nickel(II) trihydrate (2) Atomic displacement parameters (Å^2^)

**Table:**

	*U*^11^	*U*^22^	*U*^33^	*U*^12^	*U*^13^	*U*^23^
Ni1	0.02258 (16)	0.03142 (16)	0.02550 (17)	−0.00112 (9)	0.00903 (12)	−0.00065 (9)
O1	0.0284 (8)	0.0450 (9)	0.0271 (8)	−0.0037 (6)	0.0110 (6)	−0.0068 (6)
O2	0.0352 (8)	0.0542 (9)	0.0306 (8)	−0.0047 (7)	0.0188 (7)	−0.0057 (7)
O3	0.0339 (8)	0.0414 (8)	0.0331 (8)	−0.0030 (6)	0.0181 (6)	−0.0025 (6)
O4	0.0553 (10)	0.0485 (9)	0.0315 (8)	−0.0078 (8)	0.0251 (7)	−0.0083 (7)
O5	0.0433 (9)	0.0357 (8)	0.0400 (9)	0.0053 (6)	0.0197 (7)	0.0072 (6)
O6	0.0620 (12)	0.0644 (12)	0.0712 (13)	0.0334 (10)	0.0304 (10)	0.0295 (10)
O7	0.0250 (7)	0.0367 (7)	0.0355 (8)	0.0000 (6)	0.0068 (6)	0.0008 (6)
O8	0.0405 (9)	0.0359 (8)	0.0488 (10)	−0.0020 (7)	0.0162 (8)	0.0055 (7)
O9	0.0393 (9)	0.0565 (10)	0.0320 (8)	0.0068 (7)	0.0137 (7)	0.0100 (7)
O10	0.0692 (12)	0.0425 (9)	0.0553 (11)	0.0030 (8)	0.0391 (9)	0.0025 (8)
N1	0.0258 (8)	0.0270 (7)	0.0243 (8)	−0.0006 (6)	0.0101 (6)	−0.0004 (6)
N2	0.0217 (8)	0.0404 (9)	0.0219 (8)	0.0020 (7)	0.0085 (6)	0.0006 (7)
C1	0.0280 (9)	0.0277 (9)	0.0263 (9)	−0.0004 (7)	0.0115 (8)	0.0011 (7)
C2	0.0318 (10)	0.0367 (10)	0.0383 (12)	−0.0059 (8)	0.0169 (9)	−0.0017 (8)
C3	0.0303 (11)	0.0357 (10)	0.0368 (11)	−0.0096 (8)	0.0084 (9)	−0.0049 (9)
C4	0.0337 (10)	0.0307 (10)	0.0268 (10)	−0.0029 (8)	0.0053 (8)	−0.0036 (8)
C5	0.0313 (10)	0.0247 (8)	0.0233 (9)	0.0019 (7)	0.0088 (8)	0.0016 (7)
C6	0.0285 (10)	0.0311 (9)	0.0247 (9)	0.0009 (8)	0.0104 (8)	0.0003 (7)
C7	0.0363 (11)	0.0279 (9)	0.0257 (10)	0.0024 (8)	0.0130 (8)	0.0000 (7)
C8	0.0276 (10)	0.0494 (12)	0.0220 (10)	0.0069 (8)	0.0114 (8)	0.0034 (8)
C9	0.0253 (11)	0.0720 (15)	0.0267 (11)	0.0106 (10)	0.0085 (9)	0.0009 (10)
C10	0.0241 (10)	0.0720 (16)	0.0396 (13)	−0.0077 (10)	0.0101 (9)	−0.0048 (11)
C11	0.0334 (11)	0.0515 (12)	0.0357 (11)	−0.0116 (9)	0.0139 (9)	−0.0046 (10)
C12	0.0262 (9)	0.0408 (10)	0.0234 (9)	−0.0046 (8)	0.0109 (8)	−0.0020 (8)
C13	0.0424 (12)	0.0461 (12)	0.0352 (12)	0.0162 (10)	0.0211 (10)	0.0094 (9)
C14	0.0326 (10)	0.0354 (10)	0.0247 (9)	−0.0008 (8)	0.0123 (8)	−0.0007 (8)

### Bis(6-carboxypicolinato)nickel(II) trihydrate (2) Geometric parameters (Å, º)

**Table:**

Ni1—N1	1.9654 (15)	O11C—H11S	0.8386
Ni1—N2	1.9720 (16)	O11C—H11T	0.8308
Ni1—O1	2.0959 (14)	N1—C1	1.330 (2)
Ni1—O5	2.1036 (14)	N1—C5	1.335 (2)
Ni1—O3	2.1666 (14)	N2—C12	1.333 (3)
Ni1—O7	2.1940 (14)	N2—C8	1.333 (3)
O1—C6	1.265 (2)	C1—C2	1.385 (3)
O2—C6	1.243 (2)	C1—C6	1.518 (3)
O3—C7	1.253 (2)	C2—C3	1.397 (3)
O4—C7	1.271 (2)	C2—H2	0.9400
O4—H4O	0.844 (5)	C3—C4	1.386 (3)
O5—C13	1.288 (3)	C3—H3	0.9400
O6—C13	1.224 (3)	C4—C5	1.379 (3)
O7—C14	1.238 (2)	C4—H4	0.9400
O8—C14	1.284 (2)	C5—C7	1.506 (3)
O8—H8O	0.842 (5)	C8—C9	1.394 (3)
O9—H9O	0.8401	C8—C13	1.503 (3)
O9—H9P	0.8400	C9—C10	1.369 (4)
O10—H10O	0.8400	C9—H9	0.9400
O10—H10P	0.8400	C10—C11	1.393 (3)
O11A—H11O	0.8460	C10—H10	0.9400
O11A—H11P	0.8480	C11—C12	1.380 (3)
O11B—O11B^i^	1.765 (19)	C11—H11	0.9400
O11B—H11Q	0.8576	C12—C14	1.500 (3)
O11B—H11R	0.8316		
			
N1—Ni1—N2	176.53 (6)	C1—C2—H2	120.8
N1—Ni1—O1	78.84 (6)	C3—C2—H2	120.8
N2—Ni1—O1	104.49 (6)	C4—C3—C2	120.05 (18)
N1—Ni1—O5	100.48 (6)	C4—C3—H3	120.0
N2—Ni1—O5	78.52 (6)	C2—C3—H3	120.0
O1—Ni1—O5	92.69 (6)	C5—C4—C3	118.05 (18)
N1—Ni1—O3	77.29 (6)	C5—C4—H4	121.0
N2—Ni1—O3	99.40 (6)	C3—C4—H4	121.0
O1—Ni1—O3	156.09 (6)	N1—C5—C4	121.39 (17)
O5—Ni1—O3	92.85 (6)	N1—C5—C7	111.71 (16)
N1—Ni1—O7	104.37 (6)	C4—C5—C7	126.91 (17)
N2—Ni1—O7	76.60 (6)	O2—C6—O1	125.83 (18)
O1—Ni1—O7	93.18 (6)	O2—C6—C1	118.45 (17)
O5—Ni1—O7	155.12 (6)	O1—C6—C1	115.72 (16)
O3—Ni1—O7	91.51 (6)	O3—C7—O4	126.24 (19)
C6—O1—Ni1	114.51 (12)	O3—C7—C5	117.68 (16)
C7—O3—Ni1	113.04 (12)	O4—C7—C5	116.08 (17)
C7—O4—H4O	119 (2)	N2—C8—C9	120.4 (2)
C13—O5—Ni1	114.88 (13)	N2—C8—C13	113.91 (18)
C14—O7—Ni1	112.34 (12)	C9—C8—C13	125.7 (2)
C14—O8—H8O	112 (2)	C10—C9—C8	118.8 (2)
H9O—O9—H9P	102.4	C10—C9—H9	120.6
H10O—O10—H10P	103.0	C8—C9—H9	120.6
H11O—O11A—H11P	113.0	C9—C10—C11	120.4 (2)
O11B^i^—O11B—H11Q	84.6	C9—C10—H10	119.8
O11B^i^—O11B—H11R	42.2	C11—C10—H10	119.8
H11Q—O11B—H11R	126.8	C12—C11—C10	117.8 (2)
H11S—O11C—H11T	128.7	C12—C11—H11	121.1
C1—N1—C5	121.50 (16)	C10—C11—H11	121.1
C1—N1—Ni1	118.22 (13)	N2—C12—C11	121.53 (19)
C5—N1—Ni1	120.13 (12)	N2—C12—C14	111.21 (16)
C12—N2—C8	121.16 (17)	C11—C12—C14	127.26 (19)
C12—N2—Ni1	120.80 (13)	O6—C13—O5	126.3 (2)
C8—N2—Ni1	118.03 (14)	O6—C13—C8	119.0 (2)
N1—C1—C2	120.67 (17)	O5—C13—C8	114.65 (18)
N1—C1—C6	112.57 (16)	O7—C14—O8	125.42 (18)
C2—C1—C6	126.76 (17)	O7—C14—C12	119.01 (17)
C1—C2—C3	118.33 (18)	O8—C14—C12	115.57 (17)

Symmetry code: (i) −*x*+1, −*y*+2, −*z*.

### Bis(6-carboxypicolinato)nickel(II) trihydrate (2) Hydrogen-bond geometry (Å, º)

**Table:**

*D*—H···*A*	*D*—H	H···*A*	*D*···*A*	*D*—H···*A*
O4—H4*O*···O10^ii^	0.84 (1)	1.61 (1)	2.456 (2)	177 (3)
O8—H8*O*···O9^iii^	0.84 (1)	1.63 (1)	2.462 (2)	171 (3)
O9—H9*O*···O2^iv^	0.84	1.94	2.751 (2)	162
O9—H9*P*···O2^ii^	0.84	1.84	2.678 (2)	172
O10—H10*O*···O11*A*^v^	0.84	1.90	2.529 (5)	131
O10—H10*O*···O11*B*^v^	0.84	1.89	2.715 (8)	166
O10—H10*O*···O11*C*^v^	0.84	1.86	2.581 (7)	143
O10—H10*P*···O5	0.84	1.78	2.608 (2)	167
O11*A*—H11*P*···O3	0.85	2.08	2.842 (5)	149
O11*C*—H11*T*···O3	0.83	2.11	2.798 (8)	140
C2—H2···O7^iv^	0.94	2.65	3.528 (2)	155
C4—H4···O7^vi^	0.94	2.51	3.195 (2)	130
C9—H9···O1^vii^	0.94	2.55	3.275 (3)	135

Symmetry codes: (ii) *x*, −*y*+3/2, *z*−1/2; (iii) *x*, *y*+1, *z*; (iv) −*x*, *y*−1/2, −*z*+1/2; (v) *x*, −*y*+3/2, *z*+1/2; (vi) −*x*, −*y*+2, −*z*; (vii) −*x*+1, −*y*+2, −*z*+1.

### Poly[(4-chloropyridine-2,6-dicarboxylato)zinc(II)] (3) Crystal data

**Table:**

[Zn(C_7_H_2_ClNO_4_)]	*D*_x_ = 2.071 Mg m^−^^3^
*M**_r_* = 264.94	Mo *K*α radiation, λ = 0.71073 Å
Tetragonal, *P*42_1_*c*	Cell parameters from 2678 reflections
*a* = 10.0293 (5) Å	θ = 2.4–26.4°
*c* = 16.8924 (9) Å	µ = 3.19 mm^−^^1^
*V* = 1699.15 (19) Å^3^	*T* = 100 K
*Z* = 8	Block, colourless
*F*(000) = 1040	0.25 × 0.10 × 0.10 mm

### Poly[(4-chloropyridine-2,6-dicarboxylato)zinc(II)] (3) Data collection

**Table:**

Bruker APEX CCD diffractometer	2592 independent reflections
Radiation source: microsource	2266 reflections with *I* > 2σ(*I*)
Multilayer optics monochromator	*R*_int_ = 0.059
ω scans	θ_max_ = 30.7°, θ_min_ = 2.4°
Absorption correction: multi-scan SADABS (Bruker, 2008)	*h* = −13→14
*T*_min_ = 0.590, *T*_max_ = 0.746	*k* = −14→14
25321 measured reflections	*l* = −24→24

### Poly[(4-chloropyridine-2,6-dicarboxylato)zinc(II)] (3) Refinement

**Table:**

Refinement on *F*^2^	Hydrogen site location: inferred from neighbouring sites
Least-squares matrix: full	H-atom parameters constrained
*R*[*F*^2^ > 2σ(*F*^2^)] = 0.026	*w* = 1/[σ^2^(*F*_o_^2^) + (0.0248*P*)^2^ + 0.4106*P*] where *P* = (*F*_o_^2^ + 2*F*_c_^2^)/3
*wR*(*F*^2^) = 0.064	(Δ/σ)_max_ = 0.001
*S* = 1.07	Δρ_max_ = 0.42 e Å^−^^3^
2592 reflections	Δρ_min_ = −0.31 e Å^−^^3^
128 parameters	Absolute structure: Refined as an inversion twin
0 restraints	Absolute structure parameter: 0.41 (2)

### Poly[(4-chloropyridine-2,6-dicarboxylato)zinc(II)] (3) Special details

**Table:**

Geometry. All esds (except the esd in the dihedral angle between two l.s. planes) are estimated using the full covariance matrix. The cell esds are taken into account individually in the estimation of esds in distances, angles and torsion angles; correlations between esds in cell parameters are only used when they are defined by crystal symmetry. An approximate (isotropic) treatment of cell esds is used for estimating esds involving l.s. planes.
Refinement. Refined as a 2-component inversion twin.

### Poly[(4-chloropyridine-2,6-dicarboxylato)zinc(II)] (3) Fractional atomic coordinates and isotropic or equivalent isotropic displacement parameters (Å^2^)

**Table:**

	*x*	*y*	*z*	*U*_iso_*/*U*_eq_	
Zn1	0.73303 (4)	0.76705 (4)	0.46958 (2)	0.01131 (10)	
Cl1	0.73595 (8)	0.52181 (7)	0.82114 (4)	0.01753 (16)	
O1	0.9441 (2)	0.7594 (2)	0.49968 (12)	0.0137 (4)	
O2	1.0803 (2)	0.7124 (2)	0.60091 (13)	0.0148 (5)	
O3	0.5199 (2)	0.7649 (2)	0.50370 (12)	0.0169 (5)	
O4	0.3846 (2)	0.7135 (2)	0.60504 (13)	0.0154 (5)	
N1	0.7328 (3)	0.7259 (2)	0.58750 (13)	0.0109 (4)	
C1	0.8485 (3)	0.6968 (3)	0.62164 (18)	0.0104 (6)	
C2	0.8556 (3)	0.6374 (3)	0.69606 (18)	0.0127 (6)	
H2	0.9387	0.6182	0.7206	0.015*	
C3	0.7347 (3)	0.6073 (3)	0.73287 (16)	0.0130 (6)	
C4	0.6137 (3)	0.6418 (3)	0.69826 (18)	0.0132 (6)	
H4	0.5314	0.6260	0.7245	0.016*	
C5	0.6181 (3)	0.7001 (3)	0.62380 (19)	0.0123 (6)	
C6	0.9667 (3)	0.7258 (4)	0.56939 (16)	0.0115 (6)	
C7	0.4968 (3)	0.7298 (4)	0.57322 (16)	0.0128 (6)	

### Poly[(4-chloropyridine-2,6-dicarboxylato)zinc(II)] (3) Atomic displacement parameters (Å^2^)

**Table:**

	*U*^11^	*U*^22^	*U*^33^	*U*^12^	*U*^13^	*U*^23^
Zn1	0.01409 (17)	0.01086 (16)	0.00897 (16)	−0.00018 (11)	−0.00130 (12)	0.00069 (12)
Cl1	0.0218 (4)	0.0193 (3)	0.0115 (3)	−0.0007 (3)	0.0004 (3)	0.0054 (3)
O1	0.0140 (10)	0.0172 (12)	0.0098 (9)	0.0002 (9)	0.0008 (8)	0.0012 (9)
O2	0.0097 (10)	0.0218 (12)	0.0129 (10)	0.0015 (9)	0.0004 (8)	0.0013 (10)
O3	0.0125 (10)	0.0251 (13)	0.0131 (9)	0.0007 (10)	−0.0010 (8)	0.0042 (10)
O4	0.0108 (10)	0.0193 (12)	0.0162 (11)	0.0014 (9)	0.0004 (8)	0.0027 (10)
N1	0.0109 (10)	0.0113 (10)	0.0104 (10)	−0.0015 (11)	0.0006 (9)	−0.0025 (9)
C1	0.0102 (13)	0.0110 (15)	0.0100 (14)	−0.0015 (10)	0.0007 (11)	−0.0012 (11)
C2	0.0117 (14)	0.0145 (15)	0.0120 (16)	0.0005 (11)	−0.0021 (11)	0.0016 (12)
C3	0.0182 (15)	0.0128 (13)	0.0079 (12)	0.0010 (12)	−0.0010 (12)	0.0005 (10)
C4	0.0139 (15)	0.0139 (15)	0.0118 (15)	−0.0026 (11)	0.0022 (11)	−0.0016 (12)
C5	0.0121 (14)	0.0111 (16)	0.0138 (15)	−0.0009 (11)	−0.0013 (11)	−0.0020 (12)
C6	0.0100 (13)	0.0123 (14)	0.0120 (14)	−0.0005 (13)	0.0012 (10)	−0.0033 (11)
C7	0.0121 (14)	0.0121 (14)	0.0141 (13)	0.0008 (13)	−0.0020 (10)	−0.0013 (12)

### Poly[(4-chloropyridine-2,6-dicarboxylato)zinc(II)] (3) Geometric parameters (Å, º)

**Table:**

Zn1—O2^i^	1.950 (2)	O4—Zn1^iv^	1.985 (2)
Zn1—O4^ii^	1.985 (2)	N1—C1	1.329 (4)
Zn1—N1	2.034 (2)	N1—C5	1.329 (4)
Zn1—O1	2.178 (2)	C1—C2	1.393 (4)
Zn1—O3	2.214 (2)	C1—C6	1.506 (4)
Cl1—C3	1.720 (3)	C2—C3	1.395 (4)
O1—C6	1.246 (3)	C2—H2	0.9500
O2—C6	1.265 (4)	C3—C4	1.391 (5)
O2—Zn1^iii^	1.950 (2)	C4—C5	1.388 (4)
O3—C7	1.247 (3)	C4—H4	0.9500
O4—C7	1.259 (4)	C5—C7	1.516 (4)
			
O2^i^—Zn1—O4^ii^	101.71 (8)	C2—C1—C6	124.8 (3)
O2^i^—Zn1—N1	139.20 (10)	C1—C2—C3	116.8 (3)
O4^ii^—Zn1—N1	117.79 (9)	C1—C2—H2	121.6
O2^i^—Zn1—O1	105.86 (9)	C3—C2—H2	121.6
O4^ii^—Zn1—O1	102.54 (9)	C4—C3—C2	121.1 (3)
N1—Zn1—O1	76.44 (9)	C4—C3—Cl1	119.6 (2)
O2^i^—Zn1—O3	93.68 (9)	C2—C3—Cl1	119.2 (2)
O4^ii^—Zn1—O3	93.58 (9)	C5—C4—C3	117.2 (3)
N1—Zn1—O3	75.04 (9)	C5—C4—H4	121.4
O1—Zn1—O3	151.30 (7)	C3—C4—H4	121.4
C6—O1—Zn1	114.03 (18)	N1—C5—C4	121.9 (3)
C6—O2—Zn1^iii^	116.01 (18)	N1—C5—C7	113.3 (3)
C7—O3—Zn1	115.3 (2)	C4—C5—C7	124.6 (3)
C7—O4—Zn1^iv^	113.67 (19)	O1—C6—O2	126.2 (3)
C1—N1—C5	120.9 (2)	O1—C6—C1	117.6 (3)
C1—N1—Zn1	117.93 (19)	O2—C6—C1	116.2 (3)
C5—N1—Zn1	119.5 (2)	O3—C7—O4	127.2 (3)
N1—C1—C2	122.0 (3)	O3—C7—C5	115.9 (3)
N1—C1—C6	113.0 (3)	O4—C7—C5	116.8 (3)
			
C5—N1—C1—C2	0.6 (4)	Zn1—O1—C6—O2	179.1 (3)
Zn1—N1—C1—C2	−164.5 (2)	Zn1—O1—C6—C1	−0.3 (4)
C5—N1—C1—C6	176.6 (3)	Zn1^iii^—O2—C6—O1	−5.2 (5)
Zn1—N1—C1—C6	11.4 (4)	Zn1^iii^—O2—C6—C1	174.2 (2)
N1—C1—C2—C3	1.1 (5)	N1—C1—C6—O1	−7.0 (5)
C6—C1—C2—C3	−174.4 (3)	C2—C1—C6—O1	168.8 (3)
C1—C2—C3—C4	−3.2 (4)	N1—C1—C6—O2	173.5 (3)
C1—C2—C3—Cl1	174.8 (2)	C2—C1—C6—O2	−10.7 (5)
C2—C3—C4—C5	3.6 (4)	Zn1—O3—C7—O4	−175.7 (3)
Cl1—C3—C4—C5	−174.4 (2)	Zn1—O3—C7—C5	2.4 (4)
C1—N1—C5—C4	−0.2 (4)	Zn1^iv^—O4—C7—O3	5.3 (5)
Zn1—N1—C5—C4	164.7 (2)	Zn1^iv^—O4—C7—C5	−172.8 (2)
C1—N1—C5—C7	−175.4 (3)	N1—C5—C7—O3	4.8 (4)
Zn1—N1—C5—C7	−10.5 (3)	C4—C5—C7—O3	−170.2 (3)
C3—C4—C5—N1	−1.9 (5)	N1—C5—C7—O4	−176.9 (3)
C3—C4—C5—C7	172.8 (3)	C4—C5—C7—O4	8.1 (5)

Symmetry codes: (i) *y*, −*x*+2, −*z*+1; (ii) *y*, −*x*+1, −*z*+1; (iii) −*y*+2, *x*, −*z*+1; (iv) −*y*+1, *x*, −*z*+1.

### Poly[[(4-hydroxypyridine-2,6-dicarboxylato)zinc(II)] monohydrate] (4) Crystal data

**Table:**

[Zn(C_7_H_3_NO_5_)]·H_2_O	*D*_x_ = 2.108 Mg m^−^^3^
*M**_r_* = 529.02	Mo *K*α radiation, λ = 0.71073 Å
Tetragonal, *P*42_1_*c*	Cell parameters from 5895 reflections
*a* = 10.050 (1) Å	θ = 2.4–27.0°
*c* = 16.5060 (16) Å	µ = 2.96 mm^−^^1^
*V* = 1667.1 (4) Å^3^	*T* = 100 K
*Z* = 4	Block, brown
*F*(000) = 1056	0.16 × 0.10 × 0.09 mm

### Poly[[(4-hydroxypyridine-2,6-dicarboxylato)zinc(II)] monohydrate] (4) Data collection

**Table:**

Bruker APEX CCD diffractometer	2567 independent reflections
Radiation source: microsource	2371 reflections with *I* > 2σ(*I*)
Multilayer optics monochromator	*R*_int_ = 0.047
ω scans	θ_max_ = 30.9°, θ_min_ = 2.4°
Absorption correction: multi-scan SADABS (Bruker, 2008)	*h* = −14→14
*T*_min_ = 0.497, *T*_max_ = 0.746	*k* = −14→13
24393 measured reflections	*l* = −22→23

### Poly[[(4-hydroxypyridine-2,6-dicarboxylato)zinc(II)] monohydrate] (4) Refinement

**Table:**

Refinement on *F*^2^	Hydrogen site location: mixed
Least-squares matrix: full	H atoms treated by a mixture of independent and constrained refinement
*R*[*F*^2^ > 2σ(*F*^2^)] = 0.033	*w* = 1/[σ^2^(*F*_o_^2^) + (0.0285*P*)^2^ + 3.4402*P*] where *P* = (*F*_o_^2^ + 2*F*_c_^2^)/3
*wR*(*F*^2^) = 0.079	(Δ/σ)_max_ = 0.001
*S* = 1.07	Δρ_max_ = 0.70 e Å^−^^3^
2567 reflections	Δρ_min_ = −0.46 e Å^−^^3^
141 parameters	Absolute structure: Refined as an inversion twin
1 restraint	Absolute structure parameter: 0.47 (2)

### Poly[[(4-hydroxypyridine-2,6-dicarboxylato)zinc(II)] monohydrate] (4) Special details

**Table:**

Geometry. All esds (except the esd in the dihedral angle between two l.s. planes) are estimated using the full covariance matrix. The cell esds are taken into account individually in the estimation of esds in distances, angles and torsion angles; correlations between esds in cell parameters are only used when they are defined by crystal symmetry. An approximate (isotropic) treatment of cell esds is used for estimating esds involving l.s. planes.
Refinement. Refined as a 2-component inversion twin.

### Poly[[(4-hydroxypyridine-2,6-dicarboxylato)zinc(II)] monohydrate] (4) Fractional atomic coordinates and isotropic or equivalent isotropic displacement parameters (Å^2^)

**Table:**

	*x*	*y*	*z*	*U*_iso_*/*U*_eq_	Occ. (<1)
Zn1	0.72241 (4)	0.76135 (4)	0.46967 (3)	0.01217 (11)	
O1	0.9408 (3)	0.7580 (3)	0.49661 (16)	0.0159 (6)	
O2	1.0797 (3)	0.7043 (3)	0.59777 (18)	0.0152 (6)	
O3	0.5160 (3)	0.7585 (3)	0.50699 (16)	0.0174 (6)	
O4	0.3840 (3)	0.7027 (3)	0.61104 (18)	0.0155 (6)	
O5	0.7472 (3)	0.5500 (3)	0.81395 (18)	0.0212 (6)	
H5O	0.671 (3)	0.536 (6)	0.830 (4)	0.032*	
O6	1.0000	0.5000	0.8756 (3)	0.0222 (10)	
H6P	1.0783	0.4849	0.8604	0.027*	
O7	0.5000	0.5000	0.8808 (3)	0.0368 (14)	
H7A	0.5132	0.4348	0.9116	0.044*	0.5
H7B	0.4868	0.5652	0.9116	0.044*	0.5
N1	0.7320 (3)	0.7229 (3)	0.58928 (19)	0.0134 (6)	
C1	0.8490 (4)	0.6960 (4)	0.6229 (3)	0.0136 (7)	
C2	0.8598 (4)	0.6405 (4)	0.6996 (3)	0.0156 (8)	
H2	0.9444	0.6236	0.7231	0.019*	
C3	0.7419 (4)	0.6101 (4)	0.7413 (2)	0.0151 (7)	
C4	0.6192 (4)	0.6406 (4)	0.7060 (3)	0.0148 (8)	
H4	0.5382	0.6241	0.7338	0.018*	
C5	0.6198 (4)	0.6958 (4)	0.6292 (3)	0.0142 (7)	
C6	0.9661 (4)	0.7214 (4)	0.5678 (2)	0.0131 (7)	
C7	0.4964 (4)	0.7222 (4)	0.5787 (2)	0.0133 (7)	

### Poly[[(4-hydroxypyridine-2,6-dicarboxylato)zinc(II)] monohydrate] (4) Atomic displacement parameters (Å^2^)

**Table:**

	*U*^11^	*U*^22^	*U*^33^	*U*^12^	*U*^13^	*U*^23^
Zn1	0.0140 (2)	0.00984 (19)	0.01268 (18)	−0.00055 (14)	−0.00052 (16)	0.00011 (16)
O1	0.0134 (12)	0.0201 (16)	0.0143 (12)	−0.0001 (11)	−0.0004 (9)	0.0031 (12)
O2	0.0103 (12)	0.0185 (15)	0.0168 (13)	0.0002 (10)	0.0004 (10)	0.0012 (12)
O3	0.0148 (12)	0.0223 (16)	0.0152 (12)	0.0005 (11)	−0.0014 (9)	0.0027 (12)
O4	0.0110 (12)	0.0183 (15)	0.0171 (14)	−0.0003 (10)	0.0008 (10)	0.0002 (11)
O5	0.0140 (14)	0.0294 (15)	0.0203 (14)	−0.0017 (12)	−0.0021 (12)	0.0113 (11)
O6	0.021 (2)	0.023 (3)	0.023 (2)	0.0016 (17)	0.000	0.000
O7	0.071 (5)	0.020 (3)	0.019 (2)	−0.014 (3)	0.000	0.000
N1	0.0120 (13)	0.0099 (13)	0.0183 (14)	−0.0008 (13)	−0.0015 (11)	−0.0003 (11)
C1	0.0099 (16)	0.0137 (19)	0.0174 (19)	−0.0005 (13)	−0.0020 (14)	−0.0011 (15)
C2	0.0098 (18)	0.019 (2)	0.018 (2)	0.0002 (13)	−0.0018 (15)	−0.0006 (16)
C3	0.0139 (18)	0.0159 (17)	0.0156 (16)	0.0005 (14)	0.0011 (15)	0.0009 (13)
C4	0.0127 (18)	0.0146 (19)	0.0172 (19)	0.0010 (13)	0.0008 (14)	0.0011 (15)
C5	0.0123 (17)	0.0140 (18)	0.0165 (19)	0.0011 (13)	−0.0022 (14)	−0.0012 (15)
C6	0.0114 (16)	0.0106 (18)	0.0174 (17)	0.0020 (14)	−0.0006 (13)	−0.0019 (15)
C7	0.0140 (16)	0.0112 (18)	0.0147 (17)	0.0007 (14)	0.0013 (13)	−0.0009 (15)

### Poly[[(4-hydroxypyridine-2,6-dicarboxylato)zinc(II)] monohydrate] (4) Geometric parameters (Å, º)

**Table:**

Zn1—O2^i^	1.956 (3)	O6—H6P	0.8400
Zn1—O4^ii^	1.987 (3)	O7—H7A	0.8400
Zn1—N1	2.014 (3)	O7—H7B	0.8400
Zn1—O3	2.164 (3)	N1—C1	1.328 (5)
Zn1—O1	2.240 (3)	N1—C5	1.334 (5)
O1—C6	1.258 (5)	C1—C2	1.388 (6)
O2—C6	1.256 (5)	C1—C6	1.509 (5)
O2—Zn1^iii^	1.956 (3)	C2—C3	1.403 (6)
O3—C7	1.255 (5)	C2—H2	0.9500
O4—C7	1.265 (5)	C3—C4	1.398 (6)
O4—Zn1^iv^	1.987 (3)	C4—C5	1.383 (6)
O5—C3	1.344 (4)	C4—H4	0.9500
O5—H5O	0.82 (3)	C5—C7	1.518 (5)
			
O2^i^—Zn1—O4^ii^	102.10 (12)	N1—C1—C6	113.9 (3)
O2^i^—Zn1—N1	135.97 (12)	C2—C1—C6	123.8 (3)
O4^ii^—Zn1—N1	121.41 (12)	C1—C2—C3	118.0 (4)
O2^i^—Zn1—O3	94.78 (12)	C1—C2—H2	121.0
O4^ii^—Zn1—O3	94.93 (12)	C3—C2—H2	121.0
N1—Zn1—O3	76.38 (11)	O5—C3—C4	120.4 (4)
O2^i^—Zn1—O1	102.52 (12)	O5—C3—C2	120.1 (4)
O4^ii^—Zn1—O1	102.68 (12)	C4—C3—C2	119.5 (3)
N1—Zn1—O1	75.85 (11)	C5—C4—C3	117.8 (4)
O3—Zn1—O1	151.96 (9)	C5—C4—H4	121.1
C6—O1—Zn1	112.8 (2)	C3—C4—H4	121.1
C6—O2—Zn1^iii^	120.4 (3)	N1—C5—C4	122.5 (4)
C7—O3—Zn1	115.0 (2)	N1—C5—C7	112.6 (3)
C7—O4—Zn1^iv^	111.0 (3)	C4—C5—C7	124.7 (4)
C3—O5—H5O	109 (4)	O2—C6—O1	126.3 (4)
H7A—O7—H7B	105.5	O2—C6—C1	116.7 (3)
C1—N1—C5	120.0 (3)	O1—C6—C1	117.0 (3)
C1—N1—Zn1	119.4 (3)	O3—C7—O4	125.7 (4)
C5—N1—Zn1	118.9 (3)	O3—C7—C5	116.1 (3)
N1—C1—C2	122.1 (4)	O4—C7—C5	118.1 (3)

Symmetry codes: (i) *y*, −*x*+2, −*z*+1; (ii) *y*, −*x*+1, −*z*+1; (iii) −*y*+2, *x*, −*z*+1; (iv) −*y*+1, *x*, −*z*+1.

### Poly[[(4-hydroxypyridine-2,6-dicarboxylato)zinc(II)] monohydrate] (4) Hydrogen-bond geometry (Å, º)

**Table:**

*D*—H···*A*	*D*—H	H···*A*	*D*···*A*	*D*—H···*A*
O5—H5*O*···O7	0.82 (3)	1.94 (3)	2.765 (4)	172 (6)
O6—H6*P*···O5^v^	0.84	1.95	2.782 (4)	174
O7—H7*A*···O1^vi^	0.84	2.38	3.220 (4)	177
O7—H7*A*···O2^vi^	0.84	2.50	3.098 (3)	129
O7—H7*B*···O1^vii^	0.84	2.38	3.220 (4)	177
O7—H7*B*···O2^vii^	0.84	2.50	3.098 (3)	129

Symmetry codes: (v) −*x*+2, −*y*+1, *z*; (vi) −*x*+3/2, *y*−1/2, −*z*+3/2; (vii) *x*−1/2, −*y*+3/2, −*z*+3/2.

### Triaqua(4-chloropyridine-2,6-dicarboxylato)nickel(II) (5) Crystal data

**Table:**

[Ni(C_7_H_2_ClNO_4_)(H_2_O)_3_]	*D*_x_ = 1.950 Mg m^−^^3^
*M**_r_* = 312.30	Mo *K*α radiation, λ = 0.71073 Å
Tetragonal, *I*4_1_/*a*	Cell parameters from 2927 reflections
*a* = 9.544 (2) Å	θ = 2.3–26.2°
*c* = 23.361 (5) Å	µ = 2.10 mm^−^^1^
*V* = 2127.9 (11) Å^3^	*T* = 250 K
*Z* = 8	Rod, green
*F*(000) = 1264	0.40 × 0.25 × 0.18 mm

### Triaqua(4-chloropyridine-2,6-dicarboxylato)nickel(II) (5) Data collection

**Table:**

Bruker APEX CCD diffractometer	1127 independent reflections
Radiation source: microsource	935 reflections with *I* > 2σ(*I*)
Multilayer optics monochromator	*R*_int_ = 0.092
ω scans	θ_max_ = 26.8°, θ_min_ = 2.3°
Absorption correction: multi-scan SADABS (Bruker, 2008)	*h* = −12→11
*T*_min_ = 0.468, *T*_max_ = 0.745	*k* = −12→12
12540 measured reflections	*l* = −29→29

### Triaqua(4-chloropyridine-2,6-dicarboxylato)nickel(II) (5) Refinement

**Table:**

Refinement on *F*^2^	8 restraints
Least-squares matrix: full	Hydrogen site location: mixed
*R*[*F*^2^ > 2σ(*F*^2^)] = 0.050	H atoms treated by a mixture of independent and constrained refinement
*wR*(*F*^2^) = 0.127	*w* = 1/[σ^2^(*F*_o_^2^) + (0.0337*P*)^2^ + 17.5732*P*] where *P* = (*F*_o_^2^ + 2*F*_c_^2^)/3
*S* = 1.14	(Δ/σ)_max_ < 0.001
1127 reflections	Δρ_max_ = 0.50 e Å^−^^3^
99 parameters	Δρ_min_ = −0.51 e Å^−^^3^

### Triaqua(4-chloropyridine-2,6-dicarboxylato)nickel(II) (5) Special details

**Table:**

Geometry. All esds (except the esd in the dihedral angle between two l.s. planes) are estimated using the full covariance matrix. The cell esds are taken into account individually in the estimation of esds in distances, angles and torsion angles; correlations between esds in cell parameters are only used when they are defined by crystal symmetry. An approximate (isotropic) treatment of cell esds is used for estimating esds involving l.s. planes.

### Triaqua(4-chloropyridine-2,6-dicarboxylato)nickel(II) (5) Fractional atomic coordinates and isotropic or equivalent isotropic displacement parameters (Å^2^)

**Table:**

	*x*	*y*	*z*	*U*_iso_*/*U*_eq_	Occ. (<1)
Ni1	0.5000	0.2500	0.53529 (3)	0.0225 (3)	
Cl1	0.5000	0.2500	0.80909 (7)	0.0661 (8)	
O1	0.6275 (4)	0.4270 (4)	0.55472 (12)	0.0294 (8)	
O2B	0.667 (4)	0.574 (2)	0.6293 (4)	0.035 (6)	0.55 (8)
O2A	0.733 (6)	0.538 (4)	0.6258 (9)	0.054 (7)	0.45 (8)
O3	0.6692 (4)	0.1210 (4)	0.52847 (14)	0.0309 (8)	
H3O	0.740 (3)	0.163 (5)	0.523 (2)	0.037*	
H3P	0.668 (5)	0.068 (5)	0.5556 (17)	0.037*	
O4	0.5000	0.2500	0.4487 (2)	0.0361 (12)	
H4O	0.434 (3)	0.229 (7)	0.4299 (15)	0.043*	
N1	0.5000	0.2500	0.6198 (2)	0.0239 (11)	
C1	0.5686 (6)	0.3496 (5)	0.64751 (19)	0.0296 (11)	
C2	0.5715 (6)	0.3546 (5)	0.70670 (19)	0.0363 (12)	
H2	0.6198	0.4257	0.7264	0.044*	
C3	0.5000	0.2500	0.7356 (3)	0.0351 (16)	
C4	0.6370 (7)	0.4548 (6)	0.6076 (2)	0.0386 (13)	

### Triaqua(4-chloropyridine-2,6-dicarboxylato)nickel(II) (5) Atomic displacement parameters (Å^2^)

**Table:**

	*U*^11^	*U*^22^	*U*^33^	*U*^12^	*U*^13^	*U*^23^
Ni1	0.0320 (5)	0.0250 (5)	0.0104 (4)	0.0007 (4)	0.000	0.000
Cl1	0.136 (2)	0.0509 (13)	0.0115 (8)	−0.0140 (14)	0.000	0.000
O1	0.043 (2)	0.0322 (18)	0.0135 (14)	−0.0082 (15)	−0.0012 (13)	0.0011 (13)
O2B	0.059 (12)	0.023 (6)	0.021 (4)	−0.021 (6)	0.000 (4)	−0.005 (3)
O2A	0.075 (19)	0.051 (9)	0.036 (6)	−0.011 (13)	−0.013 (8)	−0.002 (6)
O3	0.036 (2)	0.034 (2)	0.0229 (16)	0.0024 (15)	−0.0037 (15)	−0.0010 (14)
O4	0.048 (3)	0.044 (3)	0.016 (2)	0.006 (3)	0.000	0.000
N1	0.031 (3)	0.020 (3)	0.021 (3)	0.000 (2)	0.000	0.000
C1	0.045 (3)	0.028 (2)	0.015 (2)	0.000 (2)	−0.0011 (19)	0.0004 (18)
C2	0.060 (4)	0.033 (3)	0.016 (2)	−0.008 (3)	−0.005 (2)	0.0001 (19)
C3	0.061 (5)	0.030 (4)	0.014 (3)	−0.001 (3)	0.000	0.000
C4	0.062 (4)	0.034 (3)	0.020 (2)	−0.016 (3)	−0.007 (2)	0.003 (2)

### Triaqua(4-chloropyridine-2,6-dicarboxylato)nickel(II) (5) Geometric parameters (Å, º)

**Table:**

Ni1—N1	1.975 (5)	O3—H3O	0.794 (19)
Ni1—O4	2.023 (5)	O3—H3P	0.809 (19)
Ni1—O3^i^	2.036 (4)	O4—H4O	0.794 (18)
Ni1—O3	2.036 (4)	N1—C1	1.323 (5)
Ni1—O1	2.131 (3)	N1—C1^i^	1.323 (5)
Ni1—O1^i^	2.131 (3)	C1—C2	1.384 (6)
Cl1—C3	1.717 (7)	C1—C4	1.518 (7)
O1—C4	1.267 (6)	C2—C3	1.385 (6)
O2B—C4	1.280 (13)	C2—H2	0.9400
O2A—C4	1.28 (2)	C3—C2^i^	1.385 (6)
			
N1—Ni1—O4	180.0	Ni1—O4—H4O	124 (3)
N1—Ni1—O3^i^	94.48 (9)	C1—N1—C1^i^	121.5 (6)
O4—Ni1—O3^i^	85.52 (9)	C1—N1—Ni1	119.3 (3)
N1—Ni1—O3	94.48 (9)	C1^i^—N1—Ni1	119.3 (3)
O4—Ni1—O3	85.52 (9)	N1—C1—C2	121.5 (5)
O3^i^—Ni1—O3	171.03 (19)	N1—C1—C4	112.8 (4)
N1—Ni1—O1	77.70 (8)	C2—C1—C4	125.6 (5)
O4—Ni1—O1	102.30 (8)	C1—C2—C3	116.9 (5)
O3^i^—Ni1—O1	89.43 (14)	C1—C2—H2	121.6
O3—Ni1—O1	92.48 (14)	C3—C2—H2	121.6
N1—Ni1—O1^i^	77.70 (8)	C2^i^—C3—C2	121.7 (6)
O4—Ni1—O1^i^	102.30 (8)	C2^i^—C3—Cl1	119.2 (3)
O3^i^—Ni1—O1^i^	92.48 (14)	C2—C3—Cl1	119.2 (3)
O3—Ni1—O1^i^	89.43 (14)	O1—C4—O2B	126.1 (7)
O1—Ni1—O1^i^	155.40 (16)	O1—C4—O2A	120.3 (15)
C4—O1—Ni1	114.5 (3)	O1—C4—C1	115.4 (4)
Ni1—O3—H3O	113 (4)	O2B—C4—C1	116.3 (7)
Ni1—O3—H3P	108 (4)	O2A—C4—C1	120.7 (8)
H3O—O3—H3P	117 (4)		

Symmetry code: (i) −*x*+1, −*y*+1/2, *z*.

### Triaqua(4-chloropyridine-2,6-dicarboxylato)nickel(II) (5) Hydrogen-bond geometry (Å, º)

**Table:**

*D*—H···*A*	*D*—H	H···*A*	*D*···*A*	*D*—H···*A*
O3—H3*P*···O2*B*^ii^	0.81 (2)	2.10 (3)	2.90 (3)	174 (5)
O3—H3*P*···O2*A*^ii^	0.81 (2)	1.85 (3)	2.628 (18)	162 (6)
O3—H3*O*···Cl1^iii^	0.79 (2)	2.75 (3)	3.464 (4)	150 (5)
O4—H4*O*···O2*B*^iv^	0.79 (2)	2.53 (6)	2.95 (3)	115 (5)
O4—H4*O*···O2*B*^v^	0.79 (2)	2.11 (5)	2.83 (3)	151 (6)

Symmetry codes: (ii) −*y*+5/4, *x*−3/4, −*z*+5/4; (iii) −*y*+5/4, *x*−1/4, *z*−1/4; (iv) −*x*+1, −*y*+1, −*z*+1; (v) *y*−1/4, −*x*+3/4, *z*−1/4.

### Triaqua(4-hydroxypyridine-2,6-dicarboxylato)nickel(II) 1.7-hydrate (6) Crystal data

**Table:**

[Ni(C_7_H_3_NO_5_)(H_2_O)_3_]·1.7H_2_O	*F*(000) = 1336
*M**_r_* = 324.49	*D*_x_ = 1.911 Mg m^−^^3^
Monoclinic, *C*2/*c*	Mo *K*α radiation, λ = 0.71073 Å
*a* = 14.7249 (11) Å	Cell parameters from 5539 reflections
*b* = 6.8538 (5) Å	θ = 2.8–30.1°
*c* = 22.3510 (16) Å	µ = 1.77 mm^−^^1^
β = 90.355 (1)°	*T* = 100 K
*V* = 2255.7 (3) Å^3^	Block, brown
*Z* = 8	0.33 × 0.32 × 0.12 mm

### Triaqua(4-hydroxypyridine-2,6-dicarboxylato)nickel(II) 1.7-hydrate (6) Data collection

**Table:**

Bruker APEX CCD diffractometer	3368 independent reflections
Radiation source: microsource	2963 reflections with *I* > 2σ(*I*)
Multilayer optics monochromator	*R*_int_ = 0.027
ω scans	θ_max_ = 31.0°, θ_min_ = 2.8°
Absorption correction: multi-scan SADABS (Bruker, 2008)	*h* = −21→21
*T*_min_ = 0.569, *T*_max_ = 0.746	*k* = −9→9
16693 measured reflections	*l* = −31→30

### Triaqua(4-hydroxypyridine-2,6-dicarboxylato)nickel(II) 1.7-hydrate (6) Refinement

**Table:**

Refinement on *F*^2^	12 restraints
Least-squares matrix: full	Hydrogen site location: mixed
*R*[*F*^2^ > 2σ(*F*^2^)] = 0.039	H atoms treated by a mixture of independent and constrained refinement
*wR*(*F*^2^) = 0.106	*w* = 1/[σ^2^(*F*_o_^2^) + (0.0471*P*)^2^ + 5.9483*P*] where *P* = (*F*_o_^2^ + 2*F*_c_^2^)/3
*S* = 1.17	(Δ/σ)_max_ < 0.001
3368 reflections	Δρ_max_ = 0.79 e Å^−^^3^
214 parameters	Δρ_min_ = −0.52 e Å^−^^3^

### Triaqua(4-hydroxypyridine-2,6-dicarboxylato)nickel(II) 1.7-hydrate (6) Special details

**Table:**

Geometry. All esds (except the esd in the dihedral angle between two l.s. planes) are estimated using the full covariance matrix. The cell esds are taken into account individually in the estimation of esds in distances, angles and torsion angles; correlations between esds in cell parameters are only used when they are defined by crystal symmetry. An approximate (isotropic) treatment of cell esds is used for estimating esds involving l.s. planes.

### Triaqua(4-hydroxypyridine-2,6-dicarboxylato)nickel(II) 1.7-hydrate (6) Fractional atomic coordinates and isotropic or equivalent isotropic displacement parameters (Å^2^)

**Table:**

	*x*	*y*	*z*	*U*_iso_*/*U*_eq_	Occ. (<1)
Ni1	0.48005 (2)	0.75027 (4)	0.63778 (2)	0.01414 (10)	
O1	0.52712 (10)	0.7082 (2)	0.54625 (7)	0.0134 (3)	
O2	0.48457 (11)	0.7612 (2)	0.45125 (7)	0.0143 (3)	
O3	0.38945 (11)	0.8469 (2)	0.70495 (7)	0.0181 (3)	
O4A	0.27374 (15)	1.0577 (4)	0.71517 (8)	0.0234 (7)	0.904 (9)
O4B	0.2459 (12)	0.959 (3)	0.7121 (7)	0.014 (5)*	0.096 (9)
O5	0.17015 (10)	1.0753 (2)	0.48993 (7)	0.0137 (3)	
H5O	0.1287	1.1189	0.5118	0.021*	
O6	0.42492 (12)	0.4707 (2)	0.63491 (7)	0.0172 (3)	
H6O	0.441 (3)	0.418 (5)	0.6044 (12)	0.042 (10)*	
H6P	0.3683 (13)	0.475 (7)	0.639 (2)	0.057 (13)*	
O7	0.57597 (12)	0.6389 (3)	0.69216 (7)	0.0204 (3)	
H7P	0.593 (3)	0.707 (5)	0.7192 (13)	0.039 (10)*	
H7O	0.573 (3)	0.527 (3)	0.7046 (19)	0.055 (13)*	
O8	0.55505 (12)	1.0078 (3)	0.64271 (8)	0.0232 (4)	
H8P	0.6094 (13)	0.990 (6)	0.6466 (18)	0.043 (11)*	
H8O	0.548 (2)	1.071 (4)	0.6119 (11)	0.029 (9)*	
O9	0.25952 (14)	0.9505 (3)	0.83233 (8)	0.0324 (5)	
H9O	0.2616	0.8316	0.8228	0.039*	
H9P	0.2564	1.0091	0.7994	0.039*	
O10	0.5000	0.2818 (8)	0.7500	0.0378 (14)	0.602 (7)
O11	0.5818 (4)	0.2770 (7)	0.7227 (2)	0.0269 (15)	0.398 (7)
H11O	0.624 (5)	0.268 (12)	0.747 (3)	0.032*	0.398 (7)
H10O	0.536 (4)	0.210 (6)	0.731 (4)	0.20 (5)*	
N1	0.38314 (12)	0.8706 (3)	0.58926 (8)	0.0117 (3)	
C1	0.38576 (13)	0.8646 (3)	0.52962 (9)	0.0097 (3)	
C2	0.31536 (13)	0.9363 (3)	0.49499 (9)	0.0103 (3)	
H2	0.3184	0.9330	0.4526	0.012*	
C3	0.23924 (13)	1.0142 (3)	0.52399 (9)	0.0112 (4)	
C4	0.23817 (14)	1.0234 (3)	0.58684 (9)	0.0130 (4)	
H4	0.1882	1.0784	0.6075	0.016*	
C5	0.31234 (14)	0.9496 (3)	0.61747 (9)	0.0129 (4)	
C6	0.47189 (14)	0.7704 (3)	0.50617 (9)	0.0109 (4)	
C7	0.32457 (16)	0.9490 (4)	0.68510 (10)	0.0194 (4)	

### Triaqua(4-hydroxypyridine-2,6-dicarboxylato)nickel(II) 1.7-hydrate (6) Atomic displacement parameters (Å^2^)

**Table:**

	*U*^11^	*U*^22^	*U*^33^	*U*^12^	*U*^13^	*U*^23^
Ni1	0.01316 (15)	0.01744 (16)	0.01178 (15)	0.00301 (10)	−0.00192 (10)	−0.00028 (10)
O1	0.0100 (7)	0.0168 (7)	0.0133 (7)	0.0032 (5)	−0.0016 (5)	0.0005 (5)
O2	0.0129 (7)	0.0168 (7)	0.0133 (7)	0.0034 (5)	0.0022 (5)	0.0000 (5)
O3	0.0188 (8)	0.0238 (8)	0.0115 (7)	0.0072 (6)	−0.0021 (6)	−0.0004 (6)
O4A	0.0238 (11)	0.0332 (15)	0.0132 (9)	0.0120 (10)	0.0010 (7)	−0.0036 (8)
O5	0.0087 (6)	0.0180 (7)	0.0143 (7)	0.0050 (5)	−0.0015 (5)	−0.0006 (6)
O6	0.0181 (8)	0.0205 (8)	0.0130 (7)	0.0016 (6)	0.0017 (6)	−0.0019 (6)
O7	0.0173 (8)	0.0301 (9)	0.0139 (7)	0.0098 (7)	−0.0032 (6)	−0.0038 (7)
O8	0.0193 (9)	0.0253 (9)	0.0248 (9)	−0.0030 (7)	−0.0096 (7)	0.0069 (7)
O9	0.0327 (10)	0.0447 (12)	0.0198 (8)	−0.0085 (9)	−0.0005 (7)	0.0067 (8)
O10	0.056 (4)	0.031 (3)	0.026 (2)	0.000	−0.007 (2)	0.000
O11	0.034 (3)	0.019 (2)	0.028 (3)	0.0041 (19)	−0.006 (2)	0.0017 (17)
N1	0.0108 (8)	0.0135 (8)	0.0108 (7)	0.0011 (6)	−0.0002 (6)	0.0007 (6)
C1	0.0068 (8)	0.0108 (8)	0.0115 (8)	−0.0001 (6)	0.0001 (6)	−0.0006 (7)
C2	0.0094 (8)	0.0109 (8)	0.0106 (8)	0.0005 (7)	−0.0008 (6)	−0.0001 (7)
C3	0.0083 (8)	0.0109 (8)	0.0142 (9)	−0.0003 (7)	−0.0013 (7)	0.0004 (7)
C4	0.0097 (9)	0.0151 (9)	0.0142 (9)	0.0030 (7)	0.0017 (7)	0.0006 (7)
C5	0.0120 (9)	0.0155 (9)	0.0113 (8)	0.0027 (7)	0.0020 (7)	−0.0007 (7)
C6	0.0097 (8)	0.0098 (8)	0.0131 (9)	0.0000 (7)	0.0008 (7)	−0.0006 (7)
C7	0.0201 (11)	0.0259 (11)	0.0121 (9)	0.0068 (9)	0.0011 (8)	0.0001 (8)

### Triaqua(4-hydroxypyridine-2,6-dicarboxylato)nickel(II) 1.7-hydrate (6) Geometric parameters (Å, º)

**Table:**

Ni1—N1	1.9681 (17)	O8—H8P	0.814 (19)
Ni1—O7	2.0082 (17)	O8—H8O	0.820 (18)
Ni1—O6	2.0816 (18)	O9—H9O	0.8433
Ni1—O8	2.0848 (19)	O9—H9P	0.8400
Ni1—O3	2.1205 (16)	O10—H10O	0.84 (2)
Ni1—O1	2.1833 (15)	O11—H11O	0.83 (2)
O1—C6	1.279 (3)	O11—H10O	0.84 (2)
O2—C6	1.244 (3)	N1—C1	1.335 (2)
O3—C7	1.262 (3)	N1—C5	1.336 (3)
O4A—C7	1.254 (3)	C1—C2	1.380 (3)
O4B—C7	1.311 (17)	C1—C6	1.519 (3)
O5—C3	1.334 (2)	C2—C3	1.404 (3)
O5—H5O	0.8400	C2—H2	0.9500
O6—H6O	0.808 (18)	C3—C4	1.406 (3)
O6—H6P	0.841 (19)	C4—C5	1.381 (3)
O7—H7P	0.805 (19)	C4—H4	0.9500
O7—H7O	0.816 (19)	C5—C7	1.521 (3)
			
N1—Ni1—O7	175.97 (7)	H11O—O11—H10O	115 (10)
N1—Ni1—O6	95.02 (7)	C1—N1—C5	120.76 (17)
O7—Ni1—O6	86.66 (7)	C1—N1—Ni1	120.80 (13)
N1—Ni1—O8	93.23 (7)	C5—N1—Ni1	118.35 (14)
O7—Ni1—O8	85.38 (8)	N1—C1—C2	121.52 (18)
O6—Ni1—O8	170.86 (7)	N1—C1—C6	112.78 (17)
N1—Ni1—O3	78.58 (7)	C2—C1—C6	125.70 (18)
O7—Ni1—O3	97.67 (6)	C1—C2—C3	118.41 (18)
O6—Ni1—O3	93.58 (7)	C1—C2—H2	120.8
O8—Ni1—O3	91.91 (7)	C3—C2—H2	120.8
N1—Ni1—O1	76.84 (6)	O5—C3—C2	117.66 (18)
O7—Ni1—O1	106.90 (6)	O5—C3—C4	122.86 (18)
O6—Ni1—O1	88.60 (6)	C2—C3—C4	119.47 (18)
O8—Ni1—O1	89.44 (7)	C5—C4—C3	117.72 (18)
O3—Ni1—O1	155.42 (6)	C5—C4—H4	121.1
C6—O1—Ni1	114.09 (13)	C3—C4—H4	121.1
C7—O3—Ni1	113.73 (14)	N1—C5—C4	122.08 (18)
C3—O5—H5O	109.5	N1—C5—C7	112.32 (18)
Ni1—O6—H6O	109 (3)	C4—C5—C7	125.59 (18)
Ni1—O6—H6P	111 (3)	O2—C6—O1	125.13 (19)
H6O—O6—H6P	114 (4)	O2—C6—C1	119.50 (18)
Ni1—O7—H7P	117 (3)	O1—C6—C1	115.36 (17)
Ni1—O7—H7O	122 (3)	O4A—C7—O3	126.4 (2)
H7P—O7—H7O	108 (4)	O3—C7—O4B	122.4 (8)
Ni1—O8—H8P	113 (3)	O4A—C7—C5	117.6 (2)
Ni1—O8—H8O	110 (2)	O3—C7—C5	115.80 (19)
H8P—O8—H8O	107 (4)	O4B—C7—C5	111.0 (8)
H9O—O9—H9P	104.0		

### Triaqua(4-hydroxypyridine-2,6-dicarboxylato)nickel(II) 1.7-hydrate (6) Hydrogen-bond geometry (Å, º)

**Table:**

*D*—H···*A*	*D*—H	H···*A*	*D*···*A*	*D*—H···*A*
O5—H5*O*···O1^i^	0.84	1.79	2.624 (2)	169
O6—H6*O*···O2^ii^	0.81 (2)	2.07 (2)	2.836 (2)	158 (4)
O6—H6*O*···O5^iii^	0.81 (2)	2.66 (4)	3.130 (2)	119 (3)
O6—H6*P*···O9^iv^	0.84 (2)	2.00 (2)	2.821 (3)	167 (4)
O7—H7*P*···O3^v^	0.81 (2)	1.96 (2)	2.751 (2)	166 (4)
O7—H7*O*···O10	0.82 (2)	2.24 (3)	2.989 (5)	152 (4)
O7—H7*O*···O11	0.82 (2)	1.77 (2)	2.574 (5)	170 (5)
O8—H8*P*···O9^v^	0.81 (2)	2.00 (2)	2.811 (3)	173 (4)
O8—H8*O*···O2^vi^	0.82 (2)	1.88 (2)	2.691 (2)	170 (3)
O9—H9*O*···O4*A*^iv^	0.84	2.12	2.934 (3)	161
O9—H9*O*···O4*B*^iv^	0.84	2.67	3.51 (2)	175
O9—H9*P*···O4*A*	0.84	1.93	2.729 (3)	159
O9—H9*P*···O4*B*	0.84	1.98	2.693 (17)	142
C4—H4···O7^i^	0.95	2.55	3.456 (3)	159

Symmetry codes: (i) *x*−1/2, *y*+1/2, *z*; (ii) −*x*+1, −*y*+1, −*z*+1; (iii) −*x*+1/2, −*y*+3/2, −*z*+1; (iv) −*x*+1/2, *y*−1/2, −*z*+3/2; (v) −*x*+1, *y*, −*z*+3/2; (vi) −*x*+1, −*y*+2, −*z*+1.
