# Supplementary material for: Zn and Ni complexes of pyridine-2,6-di­carboxyl­ates: crystal field stabilization matters!
Source: Acta Crystallogr E Crystallogr Commun. 2019 May 31;75(Pt 6):903–11. doi: 10.1107/S2056989019007461 (PMC6658973; doi:10.1107/S2056989019007461)
Supplement: Supplementary file 8 [file e-75-00903-sup8.pdf]

Supporting Information:

The thermal stability of (1) was investigated in detail on a sample with an initial mass of 13.785 mg. The thermogravimetric analysis represented below indicates that decomposition occurs in two steps. The compound loses the three cocrystallized water molecules in the temperature range between 50 and 130 °C (found -1.520 mg, -11.03%; calculated -1.649 mg, -11.96%). The second step at higher temperature can most probably be associated with decarboxylation (found -4.888 mg, -35.46%; calculated -5.373 mg, -38.98%) and slow concomitant decomposition.

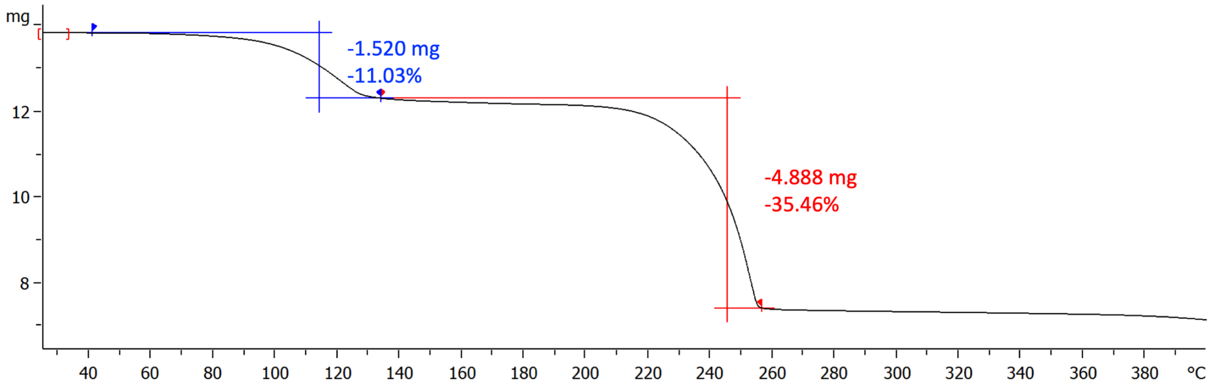

Powder patterns for (1) – (6)

Experimental diffractograms are shown in red, calculated patterns based on the single crystal diffraction experiments in black; intensities are given in arbitrary units.

(1)

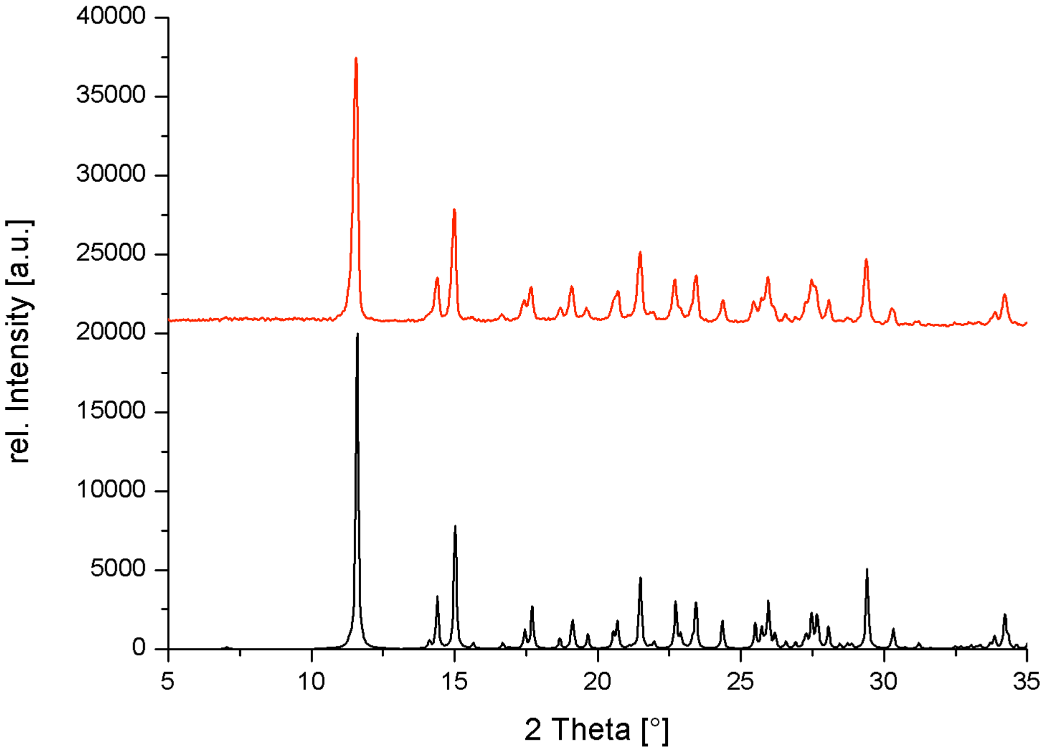

(2)

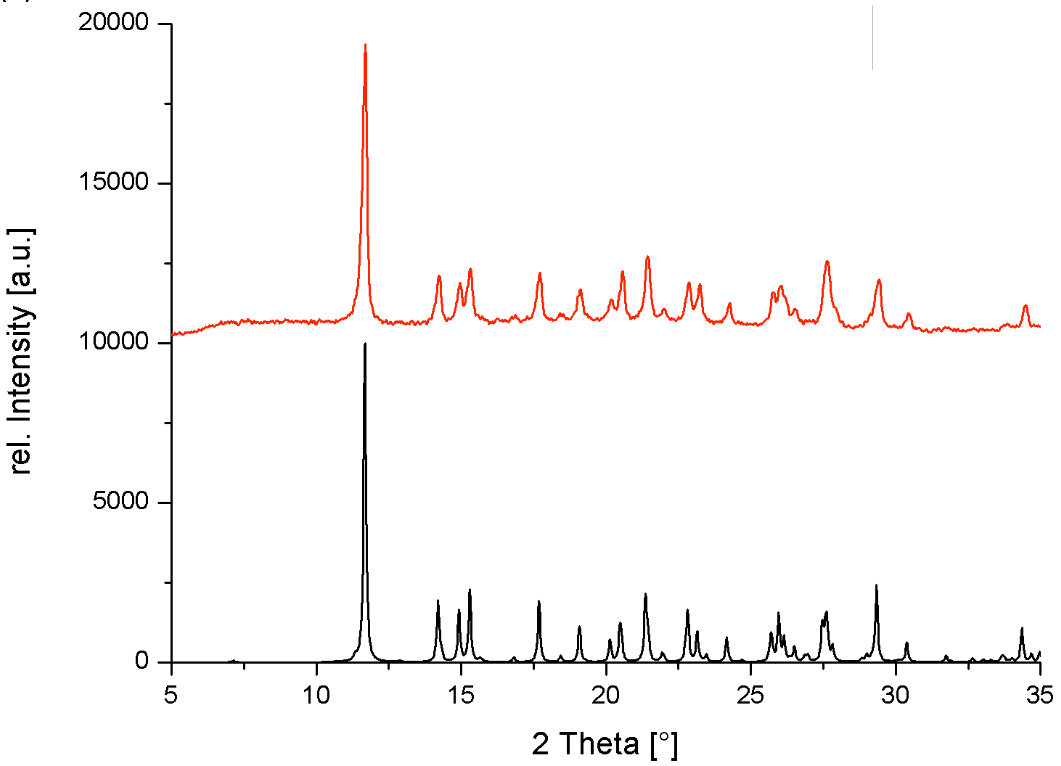

(3)

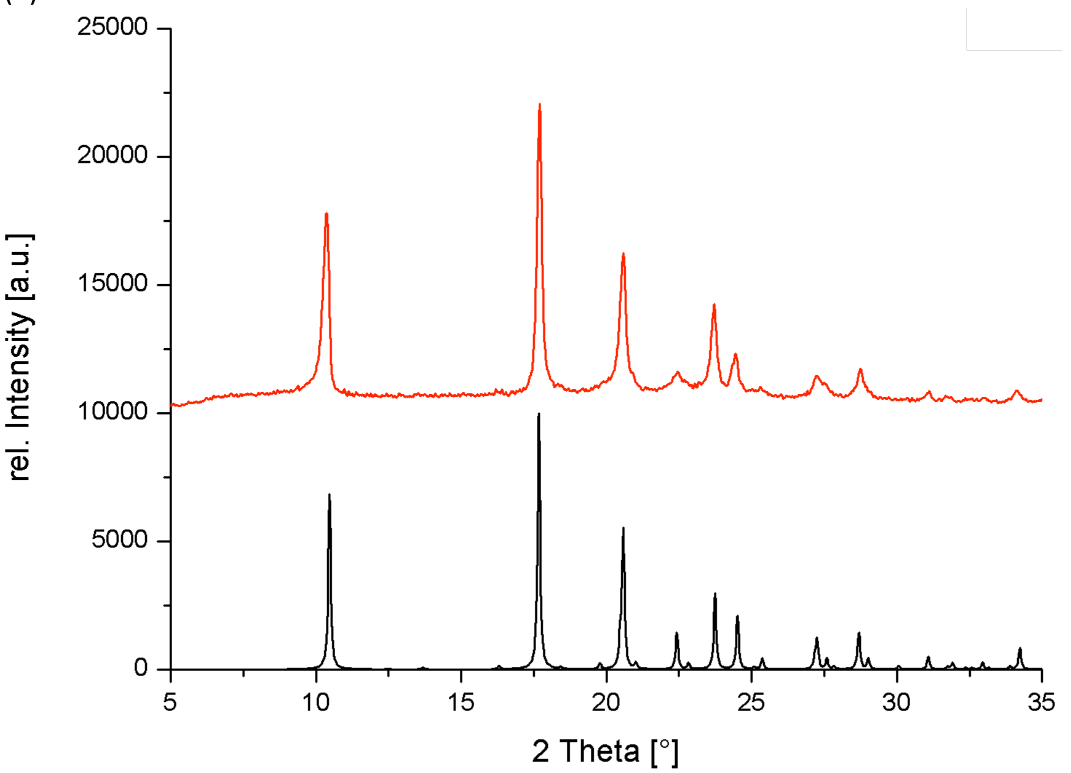
25000
20000
15000
10000
5000
0
rel. Intensity [a.u.]
5
10
15
20
25
30
35
2 Theta [°]

(4)

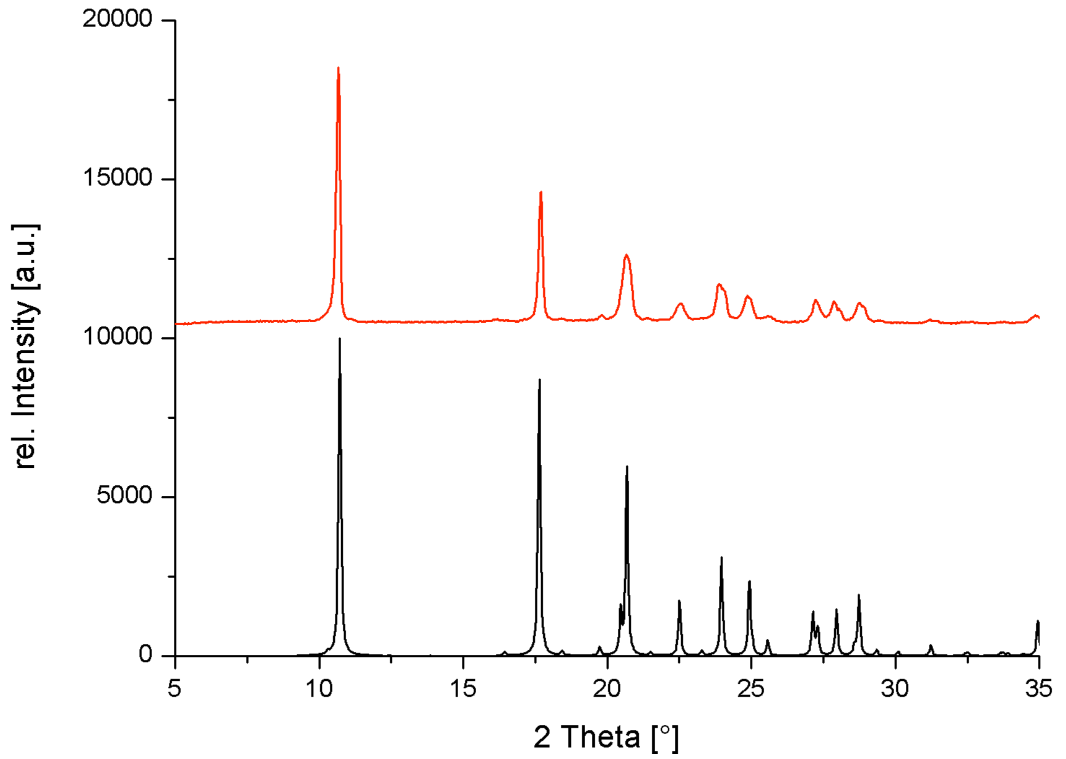
20000
15000
10000
5000
0
rel. Intensity [a.u.]
5
10
15
20
25
30
35
2 Theta [°]

(5)

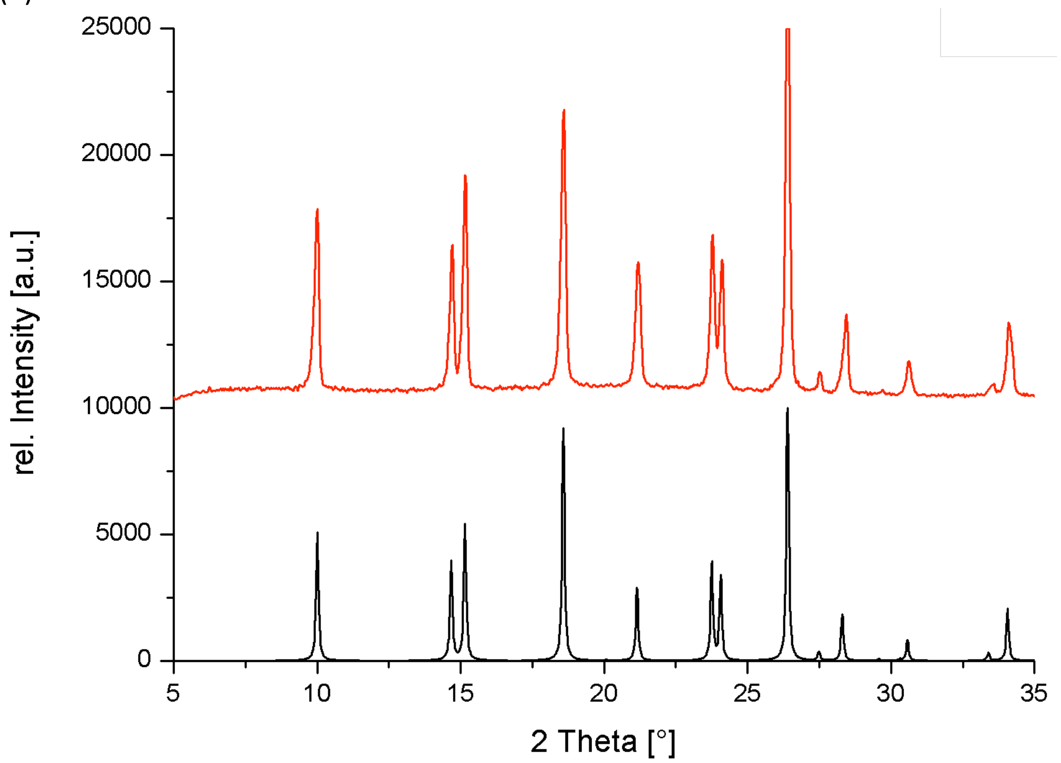

25000
20000
15000
10000
5000
0
rel. Intensity [a.u.]
5
10
15
20
25
30
35
2 Theta [°]

(6)

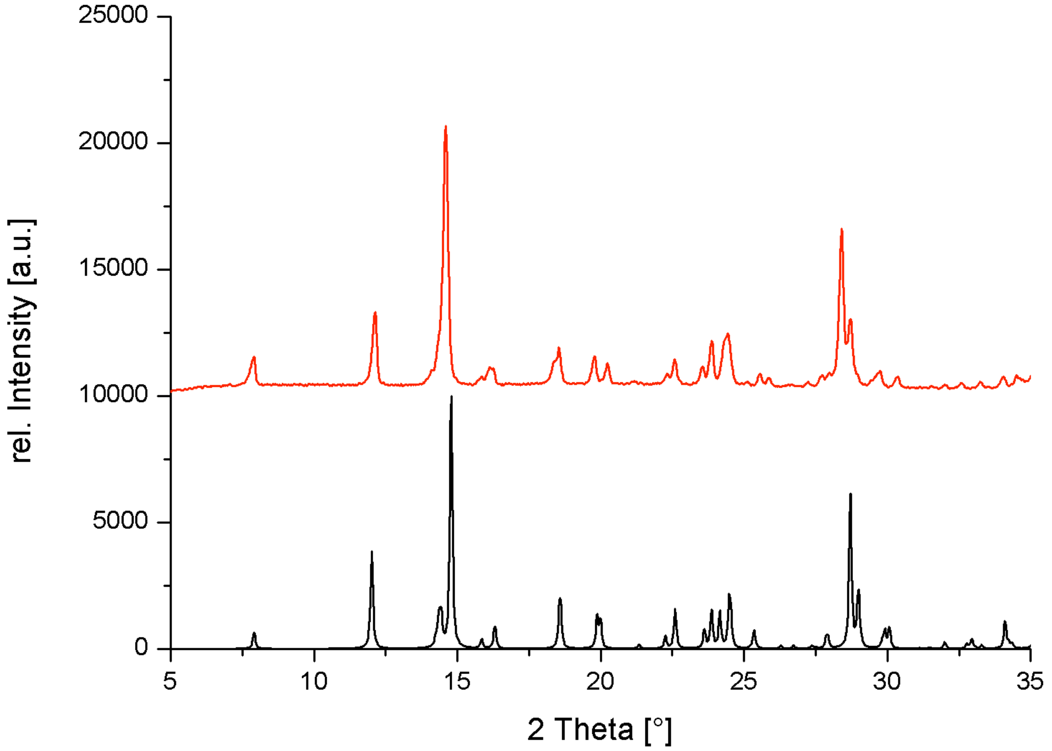

25000
20000
15000
10000
5000
0
rel. Intensity [a.u.]
5
10
15
20
25
30
35
2 Theta [°]
